# Transcriptome Analysis of *Drosophila melanogaster* Third Instar Larval Ring Glands Points to Novel Functions and Uncovers a Cytochrome p450 Required for Development

**DOI:** 10.1534/g3.116.037333

**Published:** 2016-12-13

**Authors:** Danielle Christesen, Ying Ting Yang, Jason Somers, Charles Robin, Tamar Sztal, Philip Batterham, Trent Perry

**Affiliations:** School of Biosciences, Bio21 Molecular Science and Biotechnology Institute, The University of Melbourne, Parkville, Victoria 3010, Australia

**Keywords:** ecdysteroidogenesis, immune response, cytochrome p450, Halloween genes, molting

## Abstract

In *Drosophila melanogaster* larvae, the ring gland (RG) is a control center that orchestrates major developmental transitions. It is a composite organ, consisting of the prothoracic gland, the corpus allatum, and the corpora cardiaca, each of which synthesizes and secretes a different hormone. Until now, the RG’s broader developmental roles beyond endocrine secretion have not been explored. RNA sequencing and analysis of a new transcriptome resource from *D. melanogaster* wandering third instar larval RGs has provided a fascinating insight into the diversity of developmental signaling in this organ. We have found strong enrichment of expression of two gene pathways not previously associated with the RG: immune response and fatty acid metabolism. We have also uncovered strong expression for many uncharacterized genes. Additionally, RNA interference against RG-enriched cytochrome p450s *Cyp6u1* and *Cyp6g2* produced a lethal ecdysone deficiency and a juvenile hormone deficiency, respectively, flagging a critical role for these genes in hormone synthesis. This transcriptome provides a valuable new resource for investigation of roles played by the RG in governing insect development.

Endocrine control of insect development is a complex symphony, with hormones produced in overlapping waves that determine the timing and nature of each developmental transition. In *Drosophila melanogaster* larvae, an endocrine organ, the ring gland (RG), is the control center that produces many of these hormones to orchestrate larval molts and the larval-pupal transition.

Located anterior to the larval central nervous system (CNS), the RG is a composite organ consisting of three different subtissues (King *et al.* 1966) (see Figure 1), each of which synthesizes and secretes a different hormone. The prothoracic gland (PG) is the major subtissue of the RG, both by size and cell number (King *et al.* 1966). The PG synthesizes the insect molting hormone ecdysone (Vogt 1943; Wigglesworth 1954), which is released into the hemolymph for conversion to its active form 20-hydroxyecdysone (20E) at peripheral target tissues (Petryk *et al.* 2003). 20E directly triggers major developmental events in the larva, and its precursor ecdysone is secreted by the PG cells in clearly defined pulses to provide temporal control of these events; there is a single pulse prior to each larval molt, prior to pupariation, and at the commencement of metamorphosis (Riddiford 1993; reviewed in Baehrecke 1996; Thummel 2002; Ou and King-Jones 2013).

**Figure 1:**
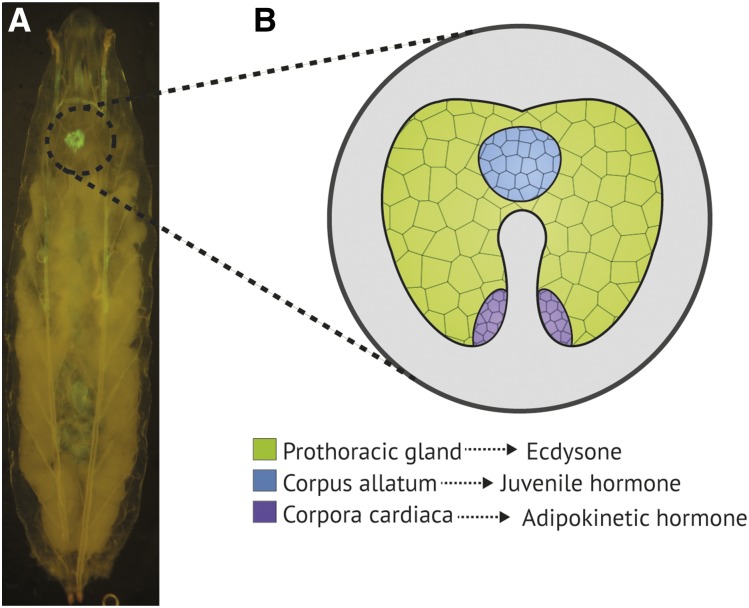
Position and substructure of the *D. melanogaster* third instar larval RG. (A) GFP expression driven by *5′phm*-GAL4 indicates the position of the RG in the whole larva. It is located dorso-anterior to the larval central nervous system. (B) The RG is a composite endocrine organ consisting of three distinct subtissues: the prothoracic gland, the corpus allatum, and the paired corpora cardiaca. Each subtissue synthesizes a different hormone.

The second-largest RG subtissue is the corpus allatum (CA) (King *et al.* 1966). Throughout the first and second larval instars, the CA cells synthesize and secrete juvenile hormone (JH), which determines the nature of all 20E-induced transitions (Williams 1961; Bownes and Rembold 1987; Sliter *et al.* 1987). In the presence of JH, 20E will always trigger a larval-larval molt (Riddiford 1970). Upon attainment of critical weight early in the third larval instar, JH production at the CA ceases, allowing 20E to initiate the changes in gene expression required for metamorphosis (reviewed in Berger and Dubrovsky 2005; Rewitz *et al.* 2013).

The third and smallest RG subtissues are the corpora cardiaca (CC), found paired at the base of each RG lobe (King *et al.* 1966). The CC cells are heavily involved in glucose regulation, being the primary site of adipokinetic hormone (Akh) production in the larva (Kim and Rulifson 2004). Akh is a peptide hormone that is functionally equivalent to mammalian glucagon; it is active in the larval fat body, where it triggers mobilization of lipids and carbohydrates into the hemolymph (Bharucha *et al.* 2008).

This transcriptome analysis of wandering third instar larvae encompasses all three RG subtissues. There are a number of questions surrounding the role of the RG subtissues that are addressed. First, there are a number of genes in the ecdysteroidogenesis pathway that are yet to be identified [known as the “Black Box” genes (reviewed in Grieneisen 1994; Rewitz *et al.* 2006; Niwa and Niwa 2014)]. Many of the known ecdysteroidogenesis reactions are catalyzed by cytochrome P450s (CYPs) (Chavez *et al.* 2000; Warren *et al.* 2002, 2004; Petryk *et al.* 2003; Niwa *et al.* 2004; Ono *et al.* 2006) so CYPs expressed in the RG would be candidate Black Box genes. Second, this transcriptomic analysis provides the opportunity to clarify ecdysteroidogenesis regulatory pathways of *D. melanogaster*. A multitude of tropic and static factors bind in the PG cells to provide tight temporal control of ecdysteroidogenesis (see Figure 2) (reviewed in Huang *et al.* 2008; Marchal *et al.* 2010; and Yamanaka *et al.* 2013); however, some components within these pathways have been investigated only in lepidoptera. Third, while ecdysteroidogenesis is recognized as the primary function of the PG, there is ultrastructural evidence from *D. melanogaster* that suggests the PG cells may be performing other roles, particularly in late larval development before the PG cells regress (Dai and Gilbert 1991).

**Figure 2:**
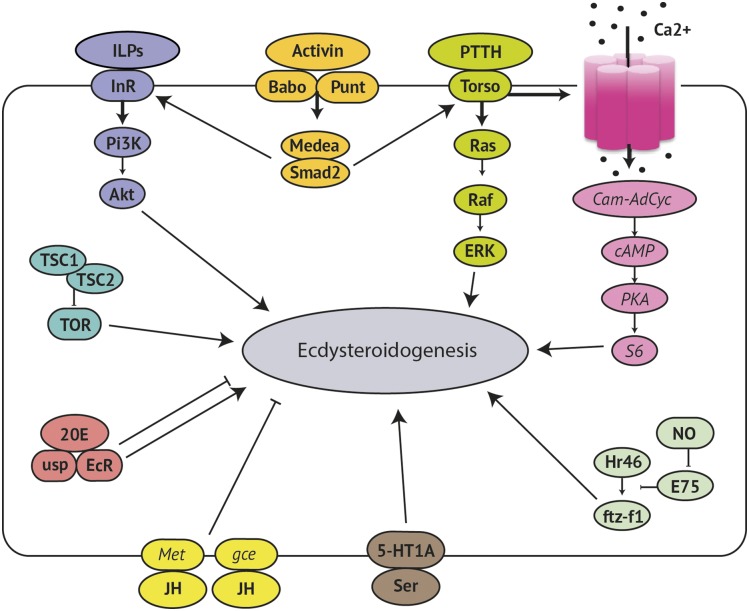
Regulation of ecdysteroidogenesis. A huge range of factors influence the ecdysteroidogenic output of the PG cells. PTTH is the major tropic regulator. When PTTH binds its receptor Torso, this activates a Ras-Raf-ERK pathway and a Ca^2+^-dependent pathway. Other tropic pathways include ILP signaling, TOR signaling, 20E signaling, serotonin signaling, and NO signaling, plus activin upregulates InR and Torso. JH and 20E can both downregulate ecdysteroidogenesis. Our knowledge of these regulatory signaling pathways comes from studies in lepidoptera only (*italic text*), or from studies in both lepidoptera and diptera (bold text). PTTH, prothoracicotropic hormone; Cam-AdCyc, calmodulin-adenylase cyclase; NO, nitric oxide; JH, juvenile hormone; 20E, 20-hydroxyecdysone; ILP, insulin-like peptide. (Adapted from Marchal *et al.* 2010; Yamanaka *et al.* 2013).

Using RNA-seq, we have gained a fresh insight into the range of genes expressed in the *D. melanogaster* wandering third instar RG. We identified 2462 genes significantly enriched in the RG relative to the CNS. As RG-enriched genes included those involved in hormone synthesis, but there were also genes involved in the immune response, and many (1310) uncharacterized genes. One of the RG-enriched CYP genes, *Cyp6u1*, was knocked down in the PG using RNA interference (RNAi). This produced a lethal low ecdysone phenotype, flagging a critical role for this gene in development. We also provide a comparison between our data and a recently published RG resource obtained by microarray (Ou *et al.* 2016). As the first complete RG transcriptome, examination of the many highly enriched genes identified in this study may ultimately reveal entirely novel function(s) of the RG subtissues.

## Materials and Methods

### Dissection, RNA isolation and sequencing

RGs were dissected from wandering third instar larvae for two wild-type strains: the reference genome strain y^1^; cn^1^ bw^1^ sp^1^ (Cel) and Armenia^14^ (A14) (Perry *et al.* 2012) (all fly stocks listed in Supplemental Material, Table S1). Dissections were performed in 100% PBS in batches of 10–40 at a time, then pooled into three biological replicates for both Cel and A14; ∼80 RGs were pooled to provide the ∼1 μg of RNA required for sequencing. Total RNA was extracted using the Reliaprep RNA Cell Miniprep System (Promega), then stored at −80°. Total RNA was quality assessed using the 2100 Bioanalyzer (Agilent Technologies), polyA enriched, cDNA libraries prepared, and 100 bp paired-end RNA-seq performed on the Illumina HiSeq2000 system (Australian Genome Research Facility, AGRF). In addition to the reads obtained from the six RG samples, duplicate RNA-seq reads for the Oregon-R wandering third instar CNS were downloaded from the modMINE database (accession: SRX029398) (Contrino *et al.* 2012). These reads were downloaded in SRA (short read archive) format, and converted to paired end fastq format using the *fastq-dump* utility included in the NCBI SRA toolkit.

### Transcriptome construction and analysis

Paired fastq sequencing reads were aligned to the annotated *D. melanogaster* reference genome (BDGP release 5) using TopHat 2.0.13 (Trapnell *et al.* 2012). Expression levels were quantified as FPKM (fragments per kilobase of transcript per million fragments mapped), and differential expression was calculated using Cufflinks 2.2.1 (Trapnell *et al.* 2012), with options to enable reference annotation based transcript assembly (–g), fragment bias correction (–b), multiread correction (–u), and increased maximum fragment alignment (–max-bundle-frags). Quality of the samples was confirmed by examining the dataset for expression of transcripts that would indicate contamination (see Figure S1). Gene ontology enrichment analysis was carried out using the Functional Annotation Clustering tool from the Database for Annotation, Visualization and Integrated Discovery (DAVID 6.7) (Huang *et al.* 2009). Clusters with enrichment scores of at least 1.3 (equivalent to nonlog *P* < 0.05) were further investigated. Secretome analysis was carried out using Signal P 4.1 (Petersen *et al.* 2011). A D-score of ≥0.45 was used as the cutoff value to discriminate signal peptides from nonsignal peptides. Flybase (St Pierre *et al.* 2014) was used to investigate gene function.

### RNAi gene knockdown

Using available UAS-dsRNA lines and DNA constructs from the Vienna Drosophila RNAi Center (VDRC) (Dietzl *et al.* 2007), select RG-enriched CYPs (*Cyp4g1*, *Cyp4d2*, *Cyp6g2*, *Cyp6u1*, and *Cyp6v1*) were knocked down. Five UAS-RNAi males were crossed to five virgin GAL4 females to achieve ubiquitous knockdown (*tubulin*-GAL4) and RG-specific knockdowns (5′*phm*-GAL4, PG; 5′*6g2*-GAL4, CA; *Akh*-GAL4, CC). All crosses were conducted at 22° with four replicates. Significance was calculated using a Student’s *t*-test. Where lethality was observed, crosses were also conducted in cages and 50 first instar larvae were picked into vials (*n* = 250). To monitor developmental timing, 10 first instar larvae were picked onto grape juice plates (*n* > 40) and developmental stages scored daily. All fly stocks used are listed in Table S1.

qPCR was used to validate RNAi knockdown of RG-enriched CYPs (*Cyp4g1*, *Cyp4d2*, *Cyp6g2*, *Cyp6u1*, and *Cyp6v1*), and to measure expression of the JH-regulated gene *Kruppel homolog 1* (*Kr-h1*) in *Cyp6g2* RNAi flies. Virgin *tubulin*-GAL4 females were crossed to males carrying each UAS-dsRNA construct and males from each of the control lines w^1118^ and 60100. For each of three biological samples, 10 whole second instar larvae were collected, and RNA was isolated using either the Reliaprep RNA Cell Miniprep System (Promega) (*Cyp4g1*, *Cyp4d2*, *Cyp6u1*, and *Cyp6v1*) or using TRIzol Reagent (Thermo Fisher Scientific) (*Cyp6g2*). RNA concentration was measured using the Qubit Fluorometer. cDNA was synthesized from 440 ng RNA using the SuperScriptIII Reverse Transcriptase kit (Invitrogen). qPCR reactions for each biological sample were carried out in triplicate using a Quanitfast SYBR Green PCR kit (Qiagen) on the CFX384 Touch Real-Time PCR Detection System (Bio-Rad). The amount of target RNA was normalized to the endogenous controls *RpL32* and *CG13220* (Van Hiel *et al.* 2009) (*Cyp4g1*, *Cyp4d2*, *Cyp6u1*, and *Cyp6v1*) or *RpL11* and *RpL24* (*Cyp6g2* and *Kr-h1*). mRNA levels were compared between samples using the ΔΔ^−Ct^ method (Bustin *et al.* 2009) using qbase+ software (Biogazelle). All primer sets used are provided in Table S2, the MIQE checklist is provided in Table S3, and qPCR results are in Figure S2.

### Ecdysteroid extraction and ELISA

Ecdysteroids were extracted and quantified following a procedure adapted from Yamanaka *et al.* (2015). Ten RG–CNS complexes were dissected and rinsed in PBS, then pooled in 300 μl of methanol on ice. The tissue was homogenized by passing through a 23-gauge needle and centrifuged at 4° for 5 min. Supernatants were collected, and the pellet re-extracted with 300 μl methanol. For hemolymph samples, 4 μl hemolymph was collected from 10 larvae, and mixed with 100 μl of methanol on ice. Samples were vortexed and centrifuged at 4° for 5 min. All supernatants were stored at −20° prior to use. Immediately prior to the ELISA, all sample solutions were dried with a SpeedVac concentrator, and dissolved in EIA buffer from the 20-hydroxyecdysone EIA kit (Cayman Chemical). The ELISA assay was performed according to the manufacturer’s instructions. Ecdysteroid levels were normalized to the amount of protein in each sample. Protein levels were measured with a Bradford Protein Assay (Bio-Rad) according to the manufacturer’s instructions.

### Data availability

Strains are available upon request. Raw sequence reads and processed data files, including the table of FPKM estimates output by Cuffdiff, are available from the National Center for Biotechnology Gene Expression Omnibus under the accession number GSE76304. 

## Results and Discussion

### Gene expression in the third instar larval ring gland

Total RNA was extracted from the RGs of Cel and A14 wandering third instar larvae then submitted for RNA-seq (summarized in Table S4). A total of 188,742,322 reads was generated by 100 bp paired-end sequencing using an Illumina Hiseq2000 at the AGRF. These reads were evenly distributed among the six samples, with the sequencing depth ranging from 28,578,817 to 33,621,275 reads. In addition to the reads obtained from the six RG samples, two replicates of RNA-seq reads for the wandering third instar CNS were downloaded from modMINE (Contrino *et al.* 2012). Given the proximity to the RG, the CNS reads were used to check for any contamination, and for differential gene expression analysis. For all eight samples, overall read alignment rates were very high, ranging from 87.1 to 91.4%. Concordant pair alignment rates were slightly lower, but still well within acceptable limits, ranging from 78.6 to 86.7%.

**Figure 3:**
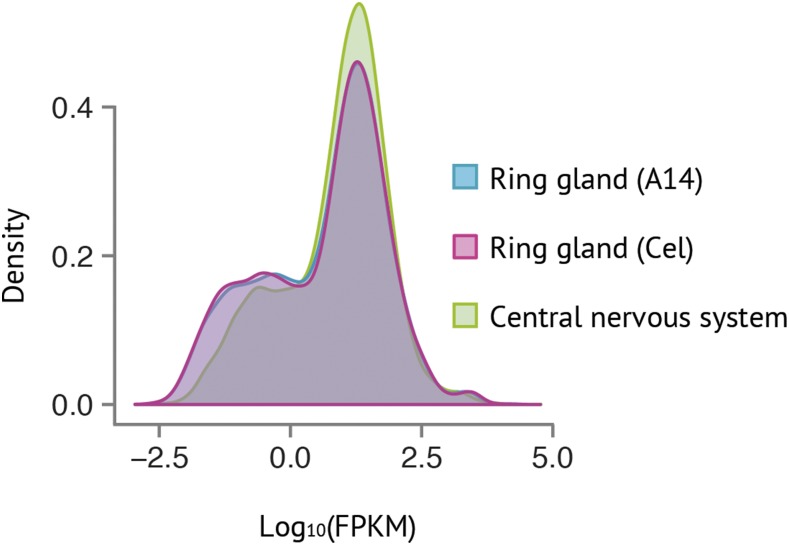
Distribution of FPKM values for all genes in the Armenia^14^ RG (blue), the Celera RG (pink), and the Oregon-R central nervous system (green). The distribution is similar for all samples. RG samples have more genes that are very lowly expressed (FPKM < 1), while the CNS has more genes that are lowly (1 < FPKM < 10) to moderately (10 < FPKM < 50) expressed. The image was generated using CummeRbund (Trapnell *et al.* 2012).

Cufflinks (Trapnell *et al.* 2012) was used to calculate the FPKM values. As can be seen in Figure 3, a similar FPKM distribution pattern was found in both RG samples and in the CNS. A large number of genes were very lowly expressed (FPKM < 1), the majority of genes were lowly (1 < FPKM < 10) to moderately expressed (10 < FPKM < 50), and there were fewer genes highly (50 < FPKM < 1000) to extremely highly (FPKM > 1000) expressed (Gelbert and Emmert 2013). Many of the genes in the latter category were ribosomal proteins (see Table S5).

To determine the total number of genes expressed in the RG, a FPKM threshold of one was applied to the dataset (Adrian and Comeron 2013; Graveley *et al.* 2011); 8292 and 8440 genes were expressed in the Cel and A14 RGs, respectively. The expression of 8055 genes was detected in both RG samples (see Table S6 for genotype-dependent RG expression). These 8055 represent 73.7% of all genes expressed in the whole body of wandering third instar larvae [10,926 genes (Daines *et al.* 2011)], a figure comparable to the number of genes expressed in the CNS (8715). Our annotation showed that 57.8% of all genes annotated in the *D. melanogaster* genome (13,918 genes, *Ensembl*, Cunningham *et al.* 2015) were expressed; 50% of genes are typically expressed in other larval tissues (Chintapalli *et al.* 2007).

Of the 8055 RG genes, differential expression analysis revealed that 2462 of these gene transcripts were significantly enriched in both RG samples relative to the CNS. The degree of enrichment exceeded 100-fold for 40 of these RG-enriched gene transcripts (see Table 1 and Table S7). Among these, 20 were genes of unknown function. The values used to calculate differential expression (CNS *vs.* RG) were from the Cel samples. Differential expression analysis using the A14 data provided similar results (see Tables S5–10, S12–13). A notable caveat of using the CNS for differential expression analysis is that transcripts may be reported as RG-enriched when in fact they are CNS-depleted relative to other tissues. This must be considered when interpreting our results.

**Table 1:** Most highly enriched genes in the RG, sorted by FPKM value

Flybase Symbol	Gene Name	FPKM*^a^*	Fold Enrichment*^a^*	GO Term*^b^*	
				Biological Process	Molecular Function
*phm*	*Phantom*	13,305	+113.35	Ecdysone biosynthetic process	Ecdysteroid 25-hydroxylase activity
*sad*	*Shadow*	12,617	+161.98	Ecdysone biosynthetic process	Ecdysteroid 2-hydroxylase activity
*Npc1a*	*Niemann-Pick C type 1a*	5479	+113.67	Regulation of cholesterol transport	*Hedgehog receptor activity*
*nvd*	*Neverland*	3759	+194.61	Ecdysteroid biosynthetic process	*Oxidoreductase activity*
*CG15919*		3706	+3370.16		
*CG4408*		3221	+123.63	*Proteolysis*	*Metallocarboxypeptidase activity*
*CG6310*		1663	+147.05		
*nobo*	*Noppera-bo*	1203	+126.97	Ecdysteroid biosynthetic process	Glutathione transferase activity
*Cyp6g2*	*Cytochrome p450 6g2*	992	+145.16	*Oxidation-reduction process*	*Monoxygenase activity*
*dib*	*Disembodied*	918	+206.62	Ecdysone biosynthetic process	Ecdysteroid 22-hydroxylase activity
*CG10337*		792	+190.80		
*CG9184*		598	+152.41		
*jhamt*	*Juvenile hormone acid methyltransferase*	587	+407.92	Juvenile hormone biosynthetic process	Juvenile hormone acid methyltransferase activity
*CG4822*		534	+122.62		*Transporter activity*
*CG6426*		524	+130.14	Multicellular organism reproduction	*Lysozyme activity*
*CG13101*		430	+202.16		
*Tsp42El*	*Tetraspanin 42El*	411	+131.97		
*CG2254*		392	+107.46	*Metabolic process*	*Oxidoreductase activity*
*Lectin-galC1*	*Galactose-specific C-type lectin*	191	+161.69	Induction of bacterial agglutination	Galactose binding
*tor*	*Torso*	162	+101.70	Metamorphosis	Protein tyrosine kinase activity
*CG30471*		120	+264.62		*Transferase activity*
*CG40006*		111	+235.07	Cell adhesion	
*Cyp6a13*	*Cytochrome p450 6a13*	107	+509.06	Defense response to bacterium	*Oxidoreductase activity*

We have selected GO terms that were most informative for our study, other GO terms for each gene can be found at FlyBase (St Pierre *et al.* 2014).

a Only Cel RG data are provided, for A14 data see Table S7.

b Regular text = based on experimental evidence, italics = based on predictions or assertions.

### Ring gland expression of ecdysteroidogenesis genes

Much of what is known about ecdysteroidogenesis comes from a combination of lepidopteran and dipteran studies. This RNA-seq data provides a more complete picture of pathways not fully investigated in *D. melanogaster*. We explored the expression levels of key genes that are either involved in the regulation of ecdysteroidogenesis, or are members of the ecdysteroidogenic pathway (see Table 2 and Table S8).

**Table 2:** Expression of select genes involved in ecdysteroidogenesis

Flybase Symbol	Gene Name	FPKM*^a^*	Fold Enrichment*^a^*	*q*-Value
Ecdysteroidogenic enzymes				
* nobo*	*Noppera-bo*	1203	+126.97	<0.001
* nvd*	*Neverland*	3759	+194.61	<0.001
* spo*	*Spook*	0.6	+15.73	0.3
* spok*	*Spookier*	0.0*^b^*	0	1
* sro*	*Shroud*	775	+59.80	<0.001
* phm*	*Phantom*	13,305	+113.35	<0.001
* dib*	*Disembodied*	918	+206.62	<0.001
* sad*	*Shadow*	12,617	+161.98	<0.001
* shd*	*Shade*	0.8	+2.68	0.09
Cholesterol homeostasis				
* Npc1a*	*Niemann Pick C type 1a*	5479	+113.67	<0.001
* Npc2a*	*Niemann Pick C type 2a*	139	+1.01	1
* Start1*	*Start1*	2277	+90.48	<0.001
* mdy*	*Midway*	129	+26.03	<0.001
PTTH signaling				
* tor*	*Torso*	162	+101.70	<0.001
* Ras*	*Ras*	97	+1.95	0.006
* Raf*	*Raf*	11	−2.11	<0.001
* ERK*	*ERK*	0.0*^b^*	0	1
* Cam*	*Calmodulin*	1240	+2.18	<0.001
* rut*	*rutabega*	18	−3.16	<0.001
* PKA*	*Protein kinase A*	71	−1.90	<0.001
* RpS6*	*Ribosomal protein S6*	2528	+1.33	0.05
* Hr4*	*Hormone receptor 4*	14	−1.35	0.02
Insulin signaling				
* InR*	*Insulin receptor*	12	−1.49	<0.001
* Pi3K*	*Phosphotidylinositol 3 kinase*	22	+1.18	0.3
* Akt*	*Akt*	50	+1.42	0.003
Activin signaling				
* babo*	*Baboon*	47	−1.13	0.4
* put*	*Punt*	67	+3.04	<0.001
* smad2/smox*	*Smad on X*	40	−2.69	<0.001
Nitric oxide signaling				
* E75*	*Ecdysone-induced protein 75*	54	−4.18	<0.001
* Hr46*	*Hormone receptor-like 46*	26	+3.07	<0.001
* ftz-f1*	*ftz transcription factor 1*	2	−1.97	0.002
TOR signaling				
* TSC1*	*TSC1*	20	−1.33	0.03
* TSC2/gig*	*TSC2*	17	+1.48	0.02
* Tor*	*Target of rapamycin*	23	+1.04	0.8
Serotonin signaling				
* 5-HT1A*	*5-hydroxytryptamine (serotonin) receptor 1A*	1	−5.70	<0.001
JH signaling				
* Met*	*Methoprene-tolerant*	4	−2.90	<0.001
* gce*	*Germ cell-expressed bHLH-PAS*	4	−2.23	<0.001
20E signaling				
* EcR*	*Ecdysone receptor*	80	+1.59	<0.001
* usp*	*Ultraspiracle*	28	−1.93	0.01

a Only Cel RG data are provided, for A14 data see Table S8.

b Genes located in heterochromatic regions were not included in reference genome. Reads corresponding to these genes were therefore not aligned by Tophat, hence the 0.0 FPKM score.

All genes in the central ecdysteroidogenesis pathway were highly expressed, with the exception of *spook* and *shade*. Low expression of *spook* was expected, given that this enzyme is required only during embryonic ecdysteroidogenesis, and not during larval stages (Ono *et al.* 2006). Low expression of *shade* is consistent with ecdysone being activated to 20E in peripheral tissues, and not the PG (Petryk *et al.* 2003). Multiple genes involved in cholesterol homeostasis were highly expressed. Cholesterol is a critical precursor for synthesis of many hormones [reviewed in Edwards and Ericsson (1999)], and the enhanced expression of *Npc1a* and *Start1* suggests that these proteins are likely the primary ER transporters responsible for cholesterol availability in the PG cells.

The enhanced expression of the prothoracicotropic hormone (PTTH) receptor, *torso*, is consistent with PTTH being the primary tropic regulator of ecdysteroidogenesis (McBrayer *et al.* 2007; Rewitz *et al.* 2009). In the tobacco hornworm *Manduca sexta*, it is clear that at least two pathways act downstream of PTTH; the Ras-Raf-ERK pathway is dominant during larval development, then, at metamorphosis, a Ca^2+^ and cAMP-dependent pathway becomes dominant (Rybczynski and Gilbert 2003) (see Figure 2). Until now, little has been noted about the Ca^2+^- and cAMP-dependent pathway in *D. melanogaster*, aside from Ca^2+^ influx appearing to be required for ecdysteroidogenesis in dissected *D. melanogaster* RGs (Henrich 1995). Here, the highly enriched expression of *Calmodulin* and *RpS6* suggests that the Ca^2+^- and cAMP-dependent branch of the PTTH pathway may be conserved in the dipteran lineage (Marchal *et al.* 2010; Lin *et al.* 2011). Another possible role for calcium signaling is regulating vesicle-mediated ecdysone release from the PG (Yamanaka *et al.* 2015). The Ca^2+^ channel/s that facilitate these two calcium-dependent pathways are yet to be identified (Fellner *et al.* 2005; Marchal *et al.* 2010), and we have detected at least nine transmembrane calcium transporters (*PMCA*, *Ca-α1T*, *pain*, *Prestin*, *Itp-r83A*, *Cac*, *Ca-α1D*, *Ca-β*, and *trp*) in the transcriptome.

All known key members of the insulin (Colombani *et al.* 2005; Caldwell *et al.* 2005; Mirth *et al.* 2005), activin (Gibbens *et al.* 2011), nitric oxide (Caceres *et al.* 2011), TOR (Layalle *et al.* 2008), and serotonin (Shimada-Niwa and Niwa 2015) pathways were also detected. The JH receptors *Met* and *gce* (Jindra *et al*. 2015) were both present, supporting evidence that JH negatively regulates ecdysone and JH synthesis at the RG (Richard and Gilbert 1991). Both components of the ecdysone receptor heterodimer, *EcR* and *usp*, were also expressed, adding to evidence that 20E is involved in feedback loops in the RG (Koelle *et al.* 1991; Karim and Thummel 1992; Song *et al.* 2003; Moeller *et al.* 2013).

### Uncharacterized cytochrome P450s are enriched in the ring gland

CYPs play an important role in the RG tissues, with the most well-known being the Halloween genes involved in ecdysteroidogenesis in the PG (Chavez *et al.* 2000; Warren *et al.* 2002, 2004; Ono *et al.* 2006). All CYPs were extracted from the dataset, and expression levels investigated to identify candidate CYPs that may belong in the Black Box, or be involved in sterol modification. CYPs that were significantly enriched in both RG samples are listed in Table 3 and Table S9. Given that developmental CYPs tend to be more highly conserved and phylogenetically stable than those involved in metabolism, the clade stability for each gene across the phylogeny of 12 *Drosophila* species was noted (Good *et al.* 2014). Any published RNAi lethality phenotypes (Chung *et al.* 2009; Guittard *et al.* 2011; Qiu *et al.* 2012) were also considered.

**Table 3:** Ring gland-enriched cytochrome p450 genes, sorted by FPKM value

Cytochrome p450	FPKM*^a^*	Fold Enrichment*^a^*	*q* Value	Annotated Biological Process*^b^*	Ubiquitous RNAi Knockdown*^c^*	Clade Stability Across *Drosophila* Species*^d^*
*phm*	13,305	+113.35	<0.001	Ecdysone biosynthetic process	Lethal	Stable
*sad*	12,617	+161.98	<0.001	Ecdysone biosynthesis process	n/a	Stable
*Cyp6g2*	992	+145.01	<0.001	*Oxidation-reduction process*	Lethal	Stable
*dib*	918	+206.62	<0.001	Ecdysone biosynthetic process	n/a	Stable
*Cyp6a13*	107	+509.06	0.007	Defense response to bacterium	Viable	Gene loss
*Cyp6v1*	96	+8.42	<0.001	*Oxidation-reduction process*	n/a	Stable
*Cyp12e1*	70	+11.96	<0.001	*Oxidation-reduction process*	Viable	Gene gain
*Cyp310a1*	52	+77.94	0.002	Negative regulation of Wnt signaling pathway	n/a	Gene loss
*Cyp6u1*	37	+3.57	<0.001	*Oxidation-reduction process*	n/a	Stable
*Cyp9f2*	20	+1.84	<0.001	Wing disc development	Viable	Gene gain
*Cyp4g1*	19	+2.72	0.08	Lipid metabolic process	Lethal	Stable
*Cyp303a1*	15	+17.58	<0.001	Sensory organ development	n/a	Stable
*Cyp4d2*	15	+3.68	<0.001	*Oxidation-reduction process*	Lethal	Gene loss
*Cyp6d4*	13	+2.36	<0.001	Wing disc development	Viable	Gene gain
*Cyp18a1*	6	+28.61	<0.001	Ecdysteroid catabolic process	Lethal	Stable

We selected GO terms that were most informative for our study; other GO terms for each gene can be found at FlyBase (St Pierre *et al.* 2014).

a Only Cel RG data are provided, for A14 data see Table S9.

b Regular text = based on experimental evidence, italics = based on predictions or assertions.

c Chung *et al.* (2009), Guittard *et al.* (2011), and Qiu *et al.* 2012.

d Good *et al.* (2014).

The most highly expressed CYPs were the known Halloween genes, plus the CA-specific *Cyp6g2*, which may be involved in JH synthesis (Chung *et al.* 2009; Wen *et al.* 2015). These genes all had expression levels >900 FPKM, but no other CYPs had expression levels in this range. Nonetheless, there were some CYPs with relevant features. *Cyp4g1* knockdown is lethal at the pupal stage (Chung *et al.* 2009; Qiu *et al.* 2012), and its closest homolog, *Bombyx mori Cyp4g25*, is induced by PTTH in the PG (Niwa *et al.* 2011). *Cyp4d2* knockdown is also lethal at the pupal stage (Chung *et al.* 2009). A notable exception from the enriched CYPs is *Cyp6t3*. Loss of *Cyp6t3* was previously shown to disrupt ecdysone biosynthesis (Ou *et al.* 2011); however, *Cyp6t3* transcripts were effectively absent from our RG samples (FPKM > 1).

#### RNAi knockdown of ring gland-enriched cytochrome p450s:

To establish whether any of the RG-enriched CYPs play an important role in the RG, we investigated their ubiquitous and RG-specific RNAi knockdown viability. A subset of the enriched CYPs (*Cyp4g1*, *Cyp4d2*, *Cyp6u1*, and *Cyp6v1*) was tested based on expression level, previously reported RNAi lethality (Chung *et al.* 2009; Guittard *et al.* 2011; Qiu *et al.* 2012), and/or the stability of their gene clade (Good *et al.* 2014).

**Figure 4:**
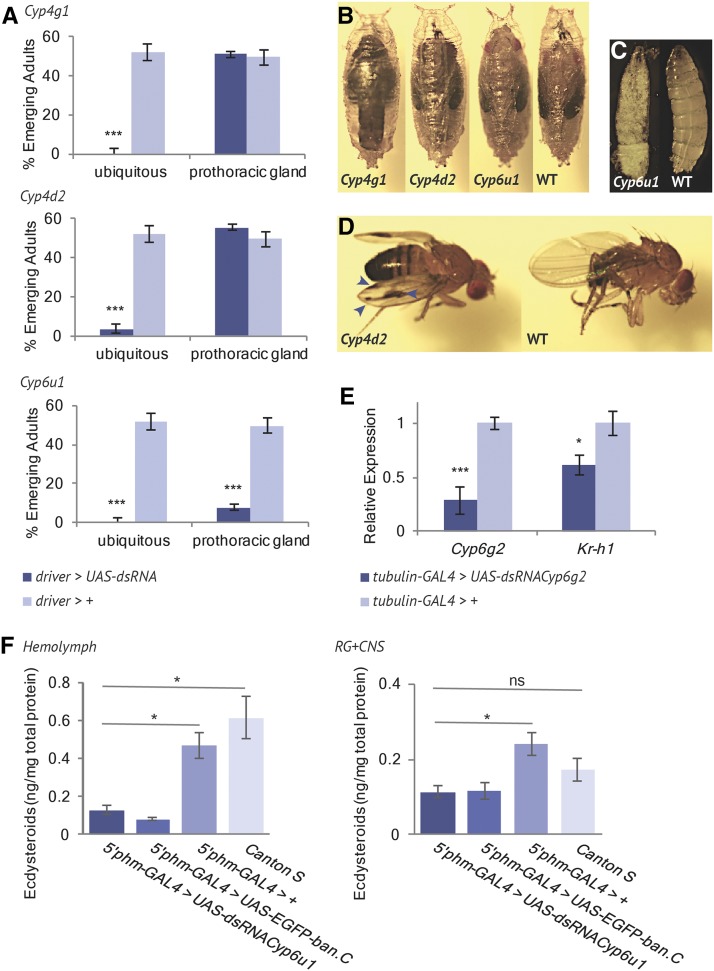
RNAi knockdown of RG-enriched cytochrome p450s. (A) Cytochrome p450s were knocked down ubiquitously (*tubulin*-GAL4), and with a PG-specific driver (5′*phm*-GAL4), and the resulting progeny scored for viability (*n* > 250). Each bar represents mean ± SEM. Significance was calculated using a Student’s *t*-test (*** *P* < 0.0001). (B, C) Representative pupae and larvae at the time of lethality and equivalent wild-type individuals. Ubiquitous knockdown is shown for *Cyp4g1* and *Cyp4d2*. PG-specific knockdown is shown for *Cyp6u1*. (D) Adults that survived ubiquitous *Cyp4d2* knockdown had variable, asymmetrical melanization on their wings (arrows). (E) qPCR reveals significantly lowered levels of *Kr-h1*, a juvenile hormone primary response gene, and *Cyp6g2* transcripts in *Cyp6g2* knockdown larvae. Significance was calculated using a Student’s *t*-test (* *P* < 0.05, *** *P* < 0.0001). (F) Quantity of ecdysteroids in the hemolymph and RG-CNS complexes of wandering third instar larvae is severely reduced in *Cyp6u1* PG-RNAi larvae. Bars represent the mean ± SEM of three independent samples. Significance was calculated using a Student’s *t*-test (* *P* < 0.05).

For *Cyp4g1*, 100% pupal lethality was observed for ubiquitous knockdown, as previously reported by Chung *et al.* (2009) and Qiu *et al.* (2012) (*n* > 250) (see Figure 4, A and B, and Figure S3). Tissue-specific knockdown of *Cyp4g1* in each of the RG subtissues had no effect on viability. We conclude that *Cyp4g1* does not play an essential developmental role in the RG, and attribute the ubiquitous knockdown lethality to *Cyp4g1*’s known role in cuticular hydrocarbon synthesis in the oenocytes (Qiu *et al.* 2012).

For *Cyp4d2*, ubiquitous RNAi resulted in 96% lethality (*n* > 250) (see Figure 4, A and B). This is consistent with the 100% pupal lethality observed by Chung *et al.* (2009), and with the EMS-induced K350X mutation that causes lethality (Haelterman *et al.* 2014). The 4% of individuals that survived to adulthood all had asymmetrical melanization on their wings (see Figure 4D). This phenotype is reminiscent of the wing patterning of *Drosophila suzukii*, and motivated us to look for this gene in the *D. suzukii* genome sequence. Interestingly, *Cyp4d2* is missing from the current *D. suzukii* genome assembly, although a short stretch of missing bases provides the possibility that the gene, by coincidence, may be present in the genome but missing in the assembly (see Figure S4). The absence of *Cyp4d2* in the *D. suzukii* transcriptome datasets (male and female) in which genes such as the Halloween CYPs are present, adds support to the proposition that *Cyp4d2* has genuinely been lost in *D. suzukii*. Wing spots in the Oriental species of the *melanogaster* species group have been gained and lost multiple times (Kopp and True 2002), and multiple loci determine their presence and size (Yeh and True 2014). *D. biarmes*, a species closely related to *D. suzukii*, has wing spots, and its genome does contain intact *Cyp4d2* coding sequence, although whether it is expressed in the relevant tissues is unknown. Thus, unexpectedly, we have stumbled on a gene that may be considered as a candidate affecting wing spots in the Oriental *Drosophila* lineage. Tissue-specific knockdown of *Cyp4d2* in each of the RG subtissues had no effect on viability (see Figure 4A and Figure S3).

**Figure 5:**
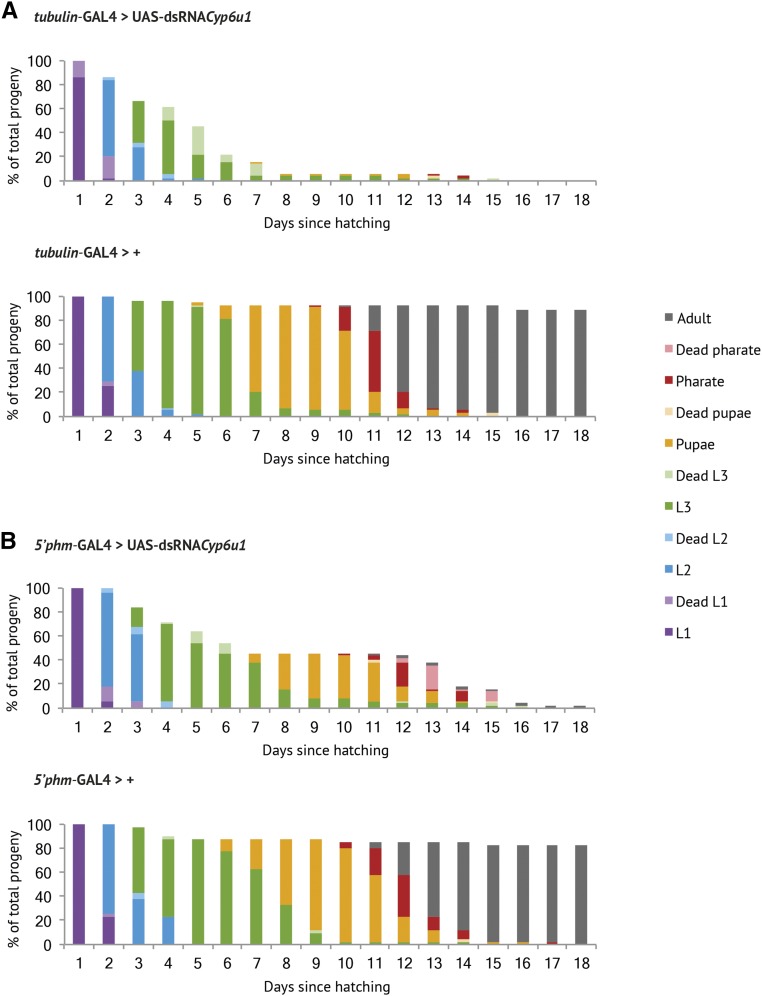
RNAi knockdown of *Cyp6u1* results in lethality throughout larval and pupal development. The percentage at each developmental stage per day posthatching (*n* > 40) is shown. Each color represents a different developmental stage, with lighter shades representing dead individuals. (A) For ubiquitous knockdown, lethality was observed at the first larval instar (32%), second larval instar (10%), and third larval instar (32%). (B) For PG-specific knockdown, lethality was observed at the first larval instar (18%), second larval instar (16%), third larval instar (26%), pupation (4%), and eclosion (34%).

For *Cyp6u1*, ubiquitous knockdown was 100% lethal (see Figure 4A) (*n* > 250), with most lethality occurring at the first larval instar (32%), second larval instar (10%), and third larval instar (54%) (*n* = 50) (see Figure 5A). Larvae often died during or shortly after molting. PG-specific knockdown of *Cyp6u1* was 92% lethal (see Figure 4, A–C), with lethality occurring at the first larval instar (18%), second larval instar (16%), third larval instar (26%), pupation (4%), and eclosion (34%) (*n* = 50) (see Figure 5B). Once again, larval lethality was often associated with incomplete moulting. This result is the first reported evidence that *Cyp6u1* may play a critical developmental role in the PG. We also quantified ecdysteroid levels in the hemolymph and RG-CNS complexes of wandering third instar *Cyp6u1* PG-specific RNAi larvae. In both the hemolymph and RG-CNS, *Cyp6u1* RNAi larvae had severely reduced ecdysteroid levels compared to the GAL4-only control, and comparable ecdysteroid levels to another ecdysone deficient line (5′*phm*-GAL4 > UAS-EGFP-ban.C; Boulan *et al.* 2013) (see Figure 4F). These low ecdysteroid levels, combined with the heterochronic lethality and incomplete molting, provide strong evidence that *Cyp6u1* may have a role in ecdysteroidogenesis, possibly in the Black Box. While the known Halloween genes all share a characteristic embryonic lethal phenotype for complete loss-of-function (Rewitz *et al.* 2006; Niwa and Niwa 2014), RNAi knockdown of Halloween genes is less severe. Individuals with PG-specific knockdown of *phm*, *dib*, or *nobo* may die as larvae or pupae (Ou *et al.* 2011; Enya *et al.* 2014). Other non-Halloween ecdysteroidogenesis genes also have similar heterochronic lethality upon PG-specific RNAi knockdown (Yoshiyama *et al.* 2006; Niwa *et al.* 2011; Ou *et al.* 2011). So far, all of our evidence suggests that *Cyp6u1* is involved in ecdysteroidogenesis, but a null allele will reveal whether complete loss-of-function is embryonic lethal, and thus whether *Cyp6u1* achieves the status of Halloween gene.

Tissue-specific knockdown of *Cyp6u1* in the CA and CC did not result in any phenotypes (see Figure S3).

For *Cyp6v1*, all ubiquitous and RG-specific knockdowns were viable (*n* > 500) (see Figure S3). qPCR indicates that *Cyp6v1* expression was actually increased in the *tubulin*-GAL4; UAS-*dsRNA-Cyp6v1* strain relative to the w^1118^; *tubulin*-GAL4 background control (see Figure S2). Thus, the RNAi knockdown was ineffective, and we are unable to report a conclusive knockdown phenotype for this gene.

#### Expression of a JH-regulated gene is decreased by Cyp6g2 RNAi:

Previous work has shown that *Cyp6g2* is the only CYP expressed in the CA, and that RNAi knockdown of *Cyp6g2* is pupal lethal (Chung *et al.* 2009). This makes *Cyp6g2* a promising candidate for the JH synthesis pathway. Using qPCR, we observed significantly reduced expression levels of *Kr-h1*, a JH primary response gene (Minakuchi *et al.* 2008; Abdou *et al.* 2011; He *et al.* 2014), in *Cyp6g2* RNAi larvae compared to GAL4-only controls (see Figure 4E). This suggests a decrease in JH titers in *Cyp6g2* RNAi larvae, and strengthens the evidence for *Cyp6g2*’s involvement in JH synthesis.

### Gene ontology enrichment analysis

Throughout the first, second, and most of the third, larval instar stages, the PG cells have a very well developed smooth endoplasmic reticulum (ER) (Dai and Gilbert 1991). This is typical of cells involved in steroid synthesis (De Loof 2008). From the wandering third instar stage, however, the smooth ER begins to regress, and the rough ER becomes abundant. This is typical of cells involved in protein secretion, suggesting the PGs may have an additional secretory role in the lead up to pupation. In this study, we have performed gene ontology enrichment analysis to uncover any genes that may be involved in any nonsteroidogenic functions. A subset of 288 transcripts was selected for this analysis using the following criteria: (1) at least 10-fold enrichment in both RG samples relative to the CNS, and (2) statistically significant enrichment in both RG samples relative to the CNS genes (see Table S10). With these 288 transcripts, we used DAVID Functional Annotation Clustering (Huang *et al.* 2009) to identify three biological processes: lipid biosynthesis, fatty acid metabolism, and immune response (see Table 4).

**Table 4:** Top ranked biological processes represented by RG-enriched transcripts

Annotation Cluster	Flybase Symbol	Gene Name
Enrichment score: 2.76		
GO:0008610 - lipid biosynthetic process	*phm*	*Phantom*
GO:0006694 - steroid biosynthetic process	*sad*	*Shadow*
GO:0008202 - steroid metabolic process	*Npc1a*	*Niemann-Pick type c 1 a*
GO:0034754 - cellular hormone metabolic process	*nvd*	*Neverland*
GO:0042446 - hormone biosynthetic process	*Start1*	*Start1*
GO:0042445 - hormone metabolic process	*dib*	*Disembodied*
GO:0010817 - regulation of hormone levels	*jhamt*	*Juvenile hormone acid methyltransferase*
GO:0042181 - ketone biosynthetic process	*hmas*	*HMG coenzyme A synthase*
GO:0045456 - ecdysteroid biosynthetic process	*CG8306*	
GO:0045455 - ecdysteroid metabolic process	*CG8239*	
GO:0016125 - sterol metabolic process	*mdy*	*Midway*
GO:0006697 - ecdysone biosynthetic process	*Pgd*	*Phosphogluconate dehydrogenase*
GO:0016126 - sterol biosynthetic process	*CG10932*	
GO:0008205 - ecdysone metabolic process	*CG8630*	
GO:0019748 - secondary metabolic process	*jheh1*	*Juvenile hormone epoxide hydrolase 1*
	*yellow-f*	*yellow-f*
	*CG17928*	
	*Cyp18a1*	*Cytochrome p450 18a1*
Enrichment score: 1.68		
GO:0006631 - fatty acid metabolic process	*CG8306*	
GO:0006633 - fatty acid biosynthetic process	*CG10932*	
GO:0016053 - organic acid biosynthetic process	*CG8630*	
GO:0046394 - carboxylic acid biosynthetic process	*CG3267*	
	*CG17928*	
	*tan*	*tan*
Enrichment score: 1.55		
GO:0019730 - antimicrobial humoral response	*Thor*	*Thor*
GO:0019731 - antibacterial humoral response	*He*	*Hemese*
GO:0009617 - response to bacterium	*TepI*	*Thioester-containing protein I*
GO:0006959 - humoral immune response	*CG16799*	
GO:0006955 - immune response	*Drs*	*Drosomycin*
GO:0042742 - defense response to bacterium	*pirk*	*poor imd response upon knock-in*
GO:0006952 - defense response	*LysS*	*Lysozyme S*
	*psh*	*Persephone*
	*TepII*	*Thioester-containing protein II*

Gene ontology enrichment analysis was carried out using the Functional Annotation Clustering tool of (DAVID 6.7) (Huang *et al.* 2009). Clusters with enrichment scores >1.3 (equivalent to nonlog *P* < 0.05) are shown.

The lipid biosynthesis cluster (18 genes) is the most significantly enriched, owing to the abundance of genes involved in ecdysone biosynthesis (*phm*, *sad*, *nvd*, and *dib*), cholesterol homeostasis (*Npc1a*, *Start1*, and *mdy*), ecdysone inactivation (*Cyp18a1*), and JH biosynthesis (*jhamt*, *hmgs*, *CG8239*, and *jheh1*). This cluster also, together with the fatty acid metabolism cluster (six genes), reveals a subset of uncharacterized genes not previously associated with any RG subtissues (*CG8360*, *CG10932*, *CG8630*, *CG17928*, and *CG3267*). These uncharacterized genes are bioinformatically predicted to modify fatty acids via branching, desaturation, or elongation. Enrichment of these genes may be associated with energy production or cholesterol storage. Another hypothesis is that the PG may use fatty acid deposits as an indicator of nutritional status to regulate ecdysteroidogenesis (Niwa *et al.* 2011). As a predicted acetoacetyl-CoA thiolase, *CG10932* may have a role in the mevalonate pathway upstream of JH biosynthesis in the CA (Bellés *et al.* 2005).

The immune response cluster (nine genes) was an unexpected finding. This cluster includes genes that actively fight microbial infection, specifically an antifungal peptide (*Drs*), and two antibacterial peptides (*LysS*, *CG16799*), plus genes that regulate the immune response. In *D. melanogaster*, the immune response is primarily orchestrated by the fat body and the hemocytes [reviewed in Hoffmann (2003)]. The cells of the fat body synthesize and secrete antimicrobial peptides upon activation of the Toll and Imd pathways (Lemaitre *et al.* 1995, 1996). The hemocytes, on the other hand, primarily participate in cellular responses such as phagocytosis, melanization, and encapsulation of parasites (Rizki and Rizki 1984), but are also capable of antimicrobial peptide production (Samakovlis *et al.* 1990). Based on this transcriptome, it is possible that the RG may be a third contributor to the immune response. The level of enrichment of immune response genes observed here would only be explained by RG expression, as we did not detect sufficient levels of fat body or hemolymph contamination (see Figure S1). In addition, we verified RG expression of a GFP-tagged immune response gene, *TepII* (see Figure S5) (Nagarkar-Jaiswal *et al*. 2015). While the *D. melanogaster* RG has not been previously associated with the immune response, proteomic analysis in the PG of the desert locust *Schistocerca gregaria* has uncovered a number of proteins involved in defense (Boerjan *et al.* 2012).

Ou *et al.* (2016) performed gene ontology enrichment analysis on an array-based RG expression dataset. They too identified “hormone biosynthesis” as a significantly enriched term; however, “immune response” and “fatty acid metabolism” were absent from their results. Inspection of their 208 RG-enriched genes reveals that only 107 were significantly enriched in our RG samples, while 65 were significantly depleted in our RG samples or had similar expression levels to the CNS (see Table S11). We attribute these differences in enrichment, and consequent differences in gene ontology results, to at least two factors; (1) the array-based subset combines four different timepoints throughout the third instar stage, so there will be transcripts specific to earlier timepoints that were not enriched in our wandering third instar samples, and (2) differential expression in the array-based dataset was calculated using whole body expression data, so relative enrichment values will differ to those calculated against CNS expression data.

### Secretome analysis of RG-enriched genes

Ultrastructural analysis of the PG cells in *D. melanogaster* has previously revealed a well-developed ER and Golgi, suggesting the PG may have a major role in protein secretion (Dai and Gilbert 1991). To identify genes containing an *N*-terminal signal peptide, the amino acid sequences of all 288 genes enriched at least 10-fold in the RG were submitted to Signal P (Petersen *et al.* 2011). Of these genes, 112 received a D-score over 0.45, and their products are therefore predicted to enter the secretory pathway, where they will either be retained at the ER, transported to the plasma membrane, or secreted from the cell (see Table S10).

The most abundant class of signal peptide-containing genes are the serine proteases (22/112 genes; 20%) (see Table 5 and Table S12). Secretion of serine proteases into the hemolymph typically initiates proteolytic cascades that then induce various innate immune responses, including melanization (Tang *et al.* 2006) and antimicrobial peptide synthesis (Ligoxygakis *et al.* 2002). A number of these RG-enriched serine proteases are known to be upregulated in response to parasitic, fungal, and bacterial infection (*Jon99Fi*, *Jon25Biii*, *CG9372*, and *CG15046*, *psh*) (Shah *et al.* 2008). There were also 26 uncharacterized genes highlighted by our analysis. *CG4408*, *CG14075*, and *CG11370* are of particular interest as they are expressed very highly, comparable to the Halloween genes *nvd*, *nobo*, and *dib* (Chavez *et al.* 2000; Yoshiyama *et al.* 2006; Enya *et al.* 2014) (see Table 1). These uncharacterized genes may represent some of the most important secreted products in the RG.

**Table 5:** RG-enriched serine proteases, sorted by FPKM value

Flybase Symbol	Gene Name	FPKM*^a^*
*CG4572*		535
*Jon99Cii*	*Jonah 99Cii*	108
*CG33465*		64
*CG33460*		63
*CG9372*		46
*CG4259*		39
*CG15046*		34
*CG10663*		22
*CG10232*		21
*CG4793*		20
*CG4927*		18
*CG4386*		18
*Jon99Fii*	*Jonah 99Fii*	16
*CG10764*		15
*Jon25Biii*	*Jonah 25Biii*	12
*CG3355*		11
*Jon99Fi*	*Jonah 99Fi*	11
*CG33225*		11
*CG33461*		9
*Jon66Cii*	*Jonah 66Cii*	7
*CG8738*		5
*psh*	*Persephone*	4

a Only Cel RG data are provided, for A14 data see Table S12.

### RG expression of immune response genes

Given the prevalence of immune response genes and serine proteases among the 288 RG-enriched genes, the RG expression levels of other key genes in the *D. melanogaster* immune system was investigated. These include genes in the two primary innate immune response pathways: the Toll pathway and the Imd pathway (see Table 6 and Table S13) [reviewed in Hoffmann (2003)]. Four of the seven genes in the Toll pathway were significantly enriched in the RG (*Myd88*, *Cactus*, *Dif*, and *Drs*). A Toll receptor (*Toll*) was expressed in both the Cel RG and the A14 RG but was significantly enriched only in the A14 RG. The Toll pathway is involved in defense against fungi and gram-positive bacteria, and activation of this pathway leads to expression of antimicrobial peptides such as drosomycin (Lemaitre *et al.* 1996). The enrichment of these Toll pathway genes suggests that the RG may have the capacity to detect fungal and gram-positive infections, and possibly contribute to the immune response by expressing antimicrobial peptides.

**Table 6:** RG expression of key genes in the immune response pathways

Flybase Symbol	Gene Name	FPKM*^a^*	Fold Enrichment*^a^*	*q*-Value
Toll Pathway				
* Tl*	*Toll*	26 (62*^b^*)	−1.46 (+1.64*^b^*)	<0.001
* Myd88*	*Myd88*	15	+1.74	<0.001
* pll*	*Pelle*	16	+1.66	0.2
* tub*	*Tube*	27	−1.19	0.2
* cact*	*Cactus*	97	+1.80	<0.001
* Dif*	*Dorsal-related immunity factor*	26	+2.71	<0.001
* Drs*	*Drosomycin*	31	+10.77	0.001
Imd Pathway				
* PGRP-LC*	*Peptidoglycan recognition protein LC*	9	+5.43	<0.001
* imd*	*Immune deficiency*	23	+1.86	0.5
* Fadd*	*Fas-associated death domain ortholog*	15	+3.06	0.1
* Dredd*	*Death related ced-3*	40	+3.74	<0.001
* Tak1*	*TGF-β activated kinase 1*	27	−1.38	0.01
* key*	*Kenny*	34	−1.19	0.3
* ird5*	*Immune response deficient 5*	16	+4.85	<0.001
* Rel*	*Relish*	30	+2.07	<0.001
* DptB*	*Diptericin*	3.5	+9.32	0.2

a Unless otherwise stated, only Cel RG data are provided. For A14 data see Table S13.

b Cel and A14 results were significantly different, therefore A14 data are provided in parentheses.

Five of the nine genes in the Imd pathway were significantly enriched in the RG (*PGRP-LC*, *Dredd*, *Tak1*, *ird5*, and *Rel*). This pathway is involved in defense against gram-negative bacteria, and its activation leads to expression of antimicrobial peptides such a diptericin (Lemaitre *et al.* 1995, 1996). The enrichment of these Imd pathway genes suggests that the RG may also be able to detect, and possibly respond to, gram-negative bacterial infections. The enrichment of these Toll and Imd pathway genes could be explained if the PG is able to use the immune status of the larva as an added level of regulation of ecdysteroidogenesis. Given that infection delays pupation (Olcott *et al.* 2010), we suggest that the PG may be able to detect an infection and then, potentially via the Toll and/or Imd pathways, directly or indirectly downregulate Halloween genes to postpone metamorphosis.

### Conclusion

This transcriptome has provided a fascinating snapshot of the diversity of developmental signaling occurring in the *D. melanogaster* third instar RG. We discovered a strong enrichment of gene pathways involved in two processes not previously associated with the *D. melanogaster* RG; immune response and fatty acid metabolism. We identified a set of enriched CYPs, at least two of which appear to be performing an essential developmental role in the RG. Furthermore, we uncovered a surplus of unnamed genes that are highly enriched, and whose characterization may help complete the ecdysone biosynthesis pathway and may even reveal additional unknown processes in the RG. Much of this transcriptome still remains to be explored. As the first complete *D. melanogaster* RG transcriptome, we hope this resource will fuel further investigations into the RG, and its broader role in governing insect development.

## Supplementary Material

Supplemental material is available online at www.g3journal.org/lookup/suppl/doi:10.1534/g3.116.037333/-/DC1.
