# Supplementary material for: Transcriptome Analysis of Drosophila melanogaster Third Instar Larval Ring Glands Points to Novel Functions and Uncovers a Cytochrome p450 Required for Development
Source: G3 (Bethesda). 2016 Dec 13;7(2):467–79. doi: 10.1534/g3.116.037333 (PMC5295594; doi:10.1534/g3.116.037333)
Supplement: Supplementary file 1 [file 467FigureS1.docx]

**
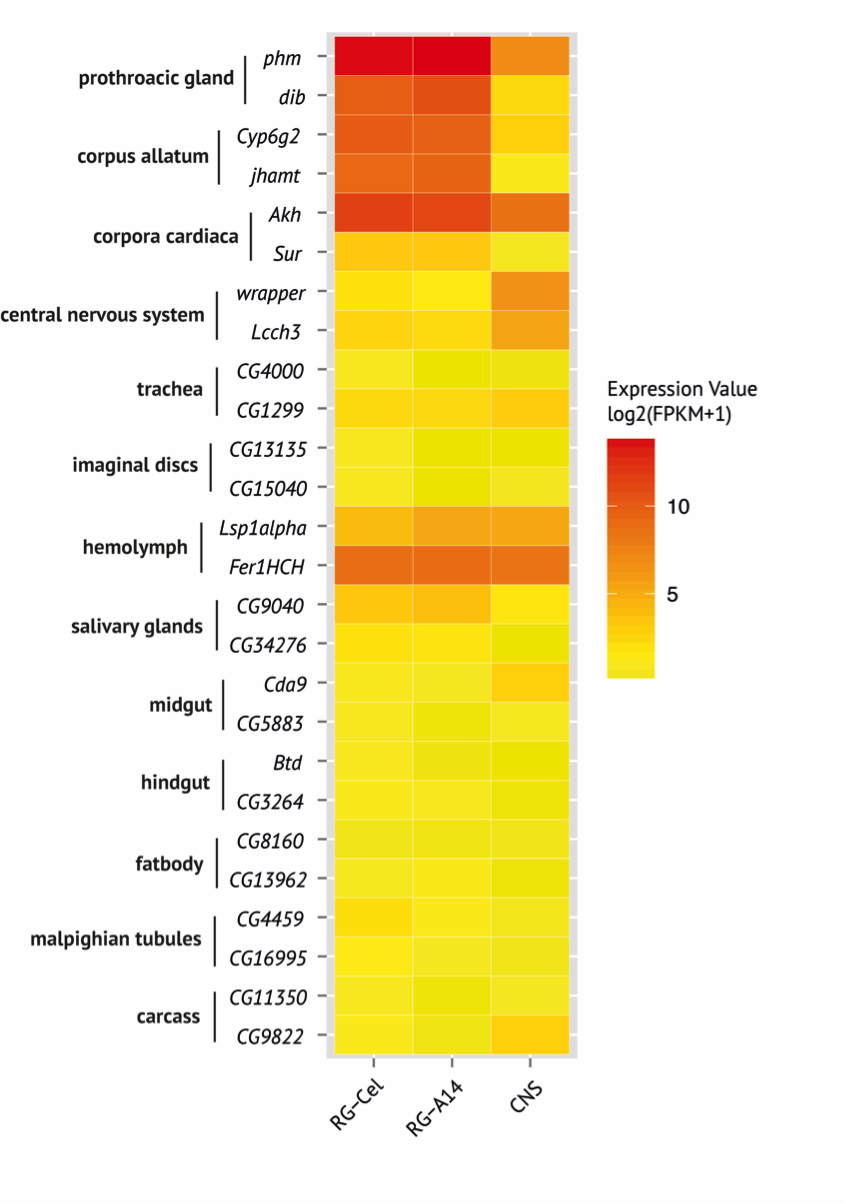
**

**Figure S1** Bioinformatic validation of the RNA-seq dataset. Shows the expression levels (log_2_FPKM) of genes highly expressed and unique to larval tissues. Genes were identified using the Flybase RNA-seq search tool (Graveley *et al.* 2011, St Pierre *et al.* 2014) and the FlyAtlas microarray expression database (Robinson *et al.* 2013).
