# Supplementary material for: Transcriptome Analysis of Drosophila melanogaster Third Instar Larval Ring Glands Points to Novel Functions and Uncovers a Cytochrome p450 Required for Development
Source: G3 (Bethesda). 2016 Dec 13;7(2):467–79. doi: 10.1534/g3.116.037333 (PMC5295594; doi:10.1534/g3.116.037333)
Supplement: Supplementary file 2 [file 467FigureS2.docx]

**
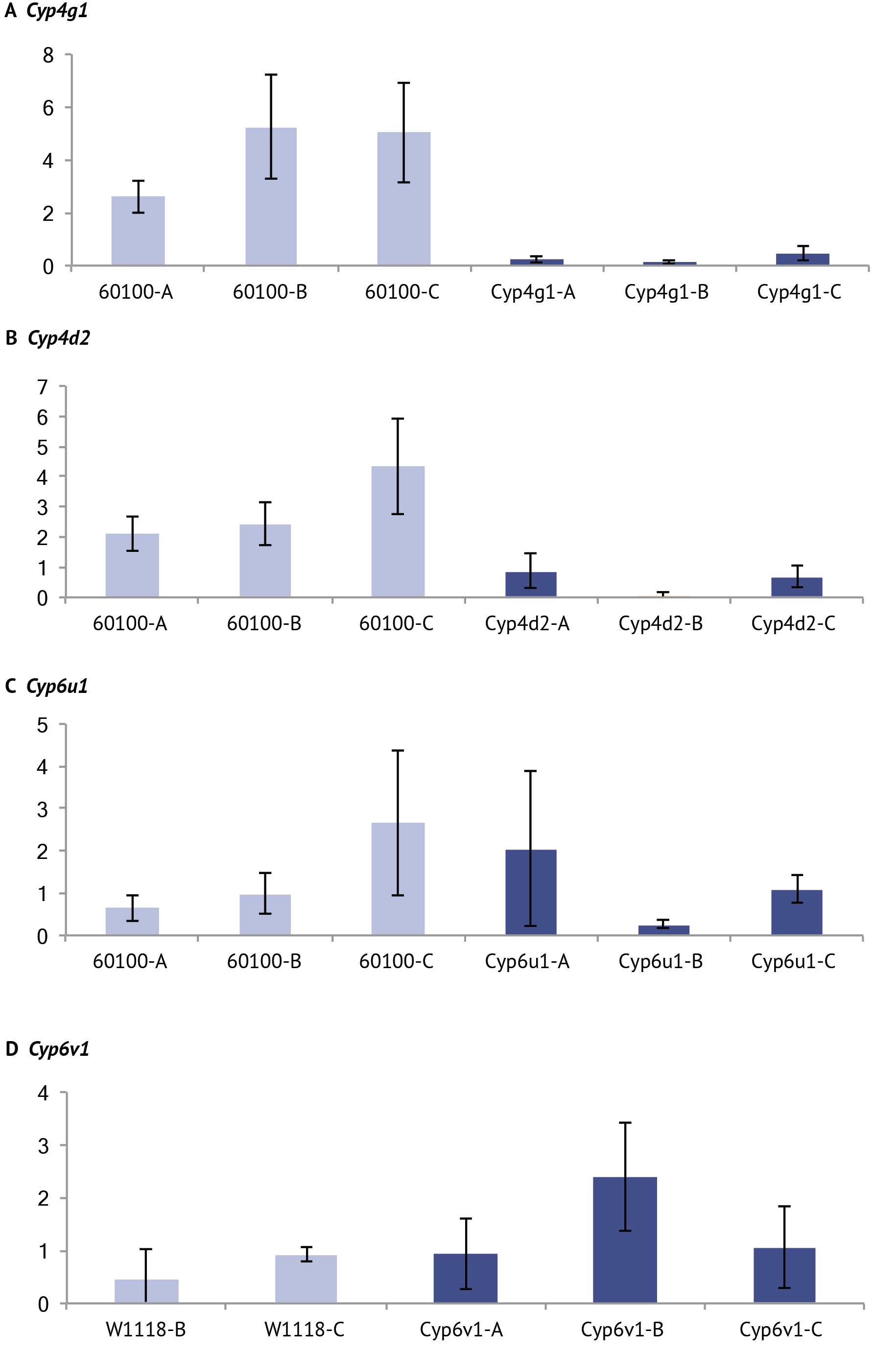
**

**Figure S2.** qPCR analysis of RNAi knockdown of ring gland-enriched cytochrome p450s. Graphs show relative expression levels between *tubulin-*GAL4>UAS-dsRNA (orange) and the tubulin-GAL4 x background control (purple).
