# Supplementary material for: Transcriptome Analysis of Drosophila melanogaster Third Instar Larval Ring Glands Points to Novel Functions and Uncovers a Cytochrome p450 Required for Development
Source: G3 (Bethesda). 2016 Dec 13;7(2):467–79. doi: 10.1534/g3.116.037333 (PMC5295594; doi:10.1534/g3.116.037333)
Supplement: Supplementary file 3 [file 467FigureS3.docx]

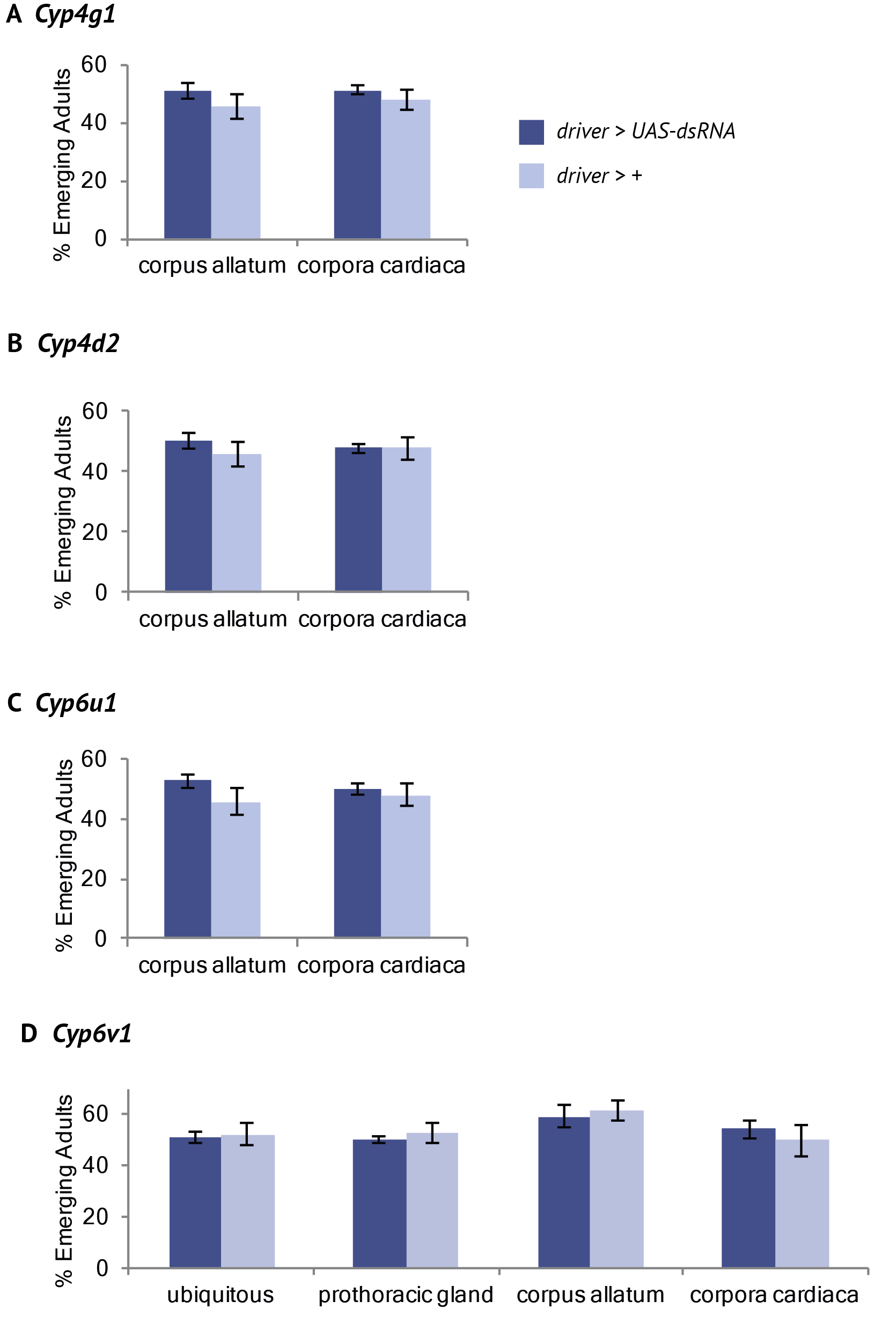


**Figure S3** RNAi knockdown of ring gland-enriched cytochrome p450s. (**A**) *Cyp4g1,* (**B**) *Cyp4d2*, (**C**) *Cyp6u1* and (**D**) *Cyp6v1* were knocked down ubiquitously (*tubulin-*GAL4), and with ring gland-specific drivers (5’*phm*-GAL4, prothoracic gland; 5’*6g2*-GAL4, corpus allatum; *Akh*-GAL4, corpora cardiaca). Significant results are presented in **Figure 4**.
