# Supplementary material for: Transcriptome Analysis of Drosophila melanogaster Third Instar Larval Ring Glands Points to Novel Functions and Uncovers a Cytochrome p450 Required for Development
Source: G3 (Bethesda). 2016 Dec 13;7(2):467–79. doi: 10.1534/g3.116.037333 (PMC5295594; doi:10.1534/g3.116.037333)
Supplement: Supplementary file 4 [file 467FigureS4.docx]

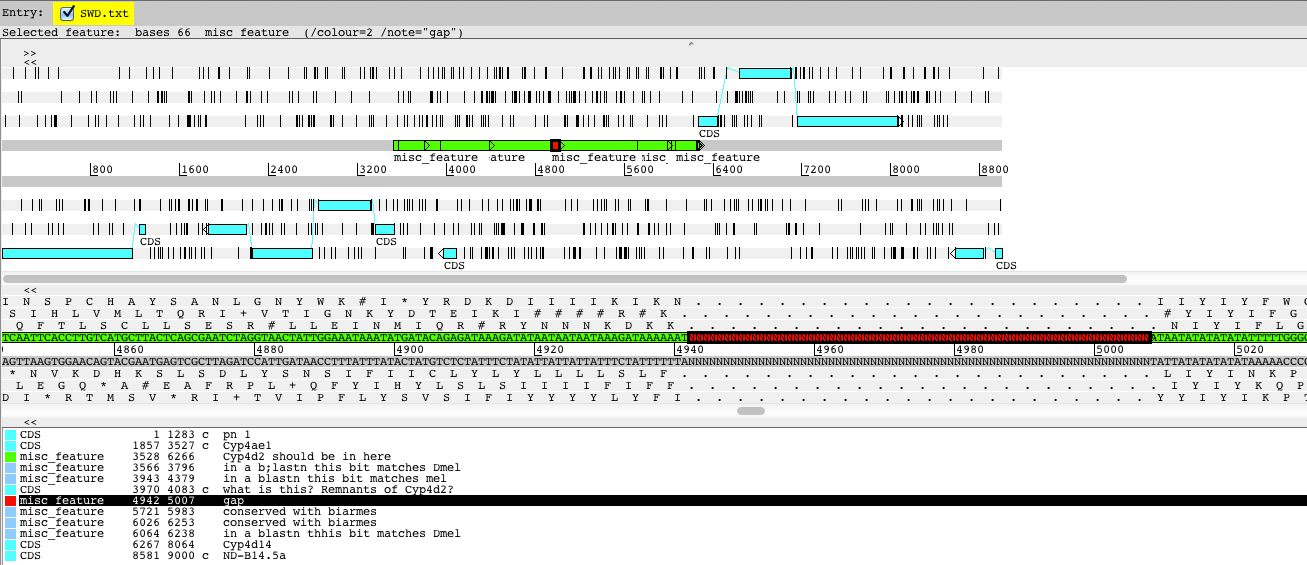


**Figure S4** Shows a screenshot of the *Drosophila suzukii* genome in Artemis, with the expected location of *Cyp4d2* in green. Whilst *Cyp4d2* is missing from the current *D. suzukii* genome assembly, there is a stretch of missing bases (red) where *Cyp4d2* may be located.
