# Supplementary material for: Transcriptome Analysis of Drosophila melanogaster Third Instar Larval Ring Glands Points to Novel Functions and Uncovers a Cytochrome p450 Required for Development
Source: G3 (Bethesda). 2016 Dec 13;7(2):467–79. doi: 10.1534/g3.116.037333 (PMC5295594; doi:10.1534/g3.116.037333)
Supplement: Supplementary file 5 [file 467FigureS5.docx]

**
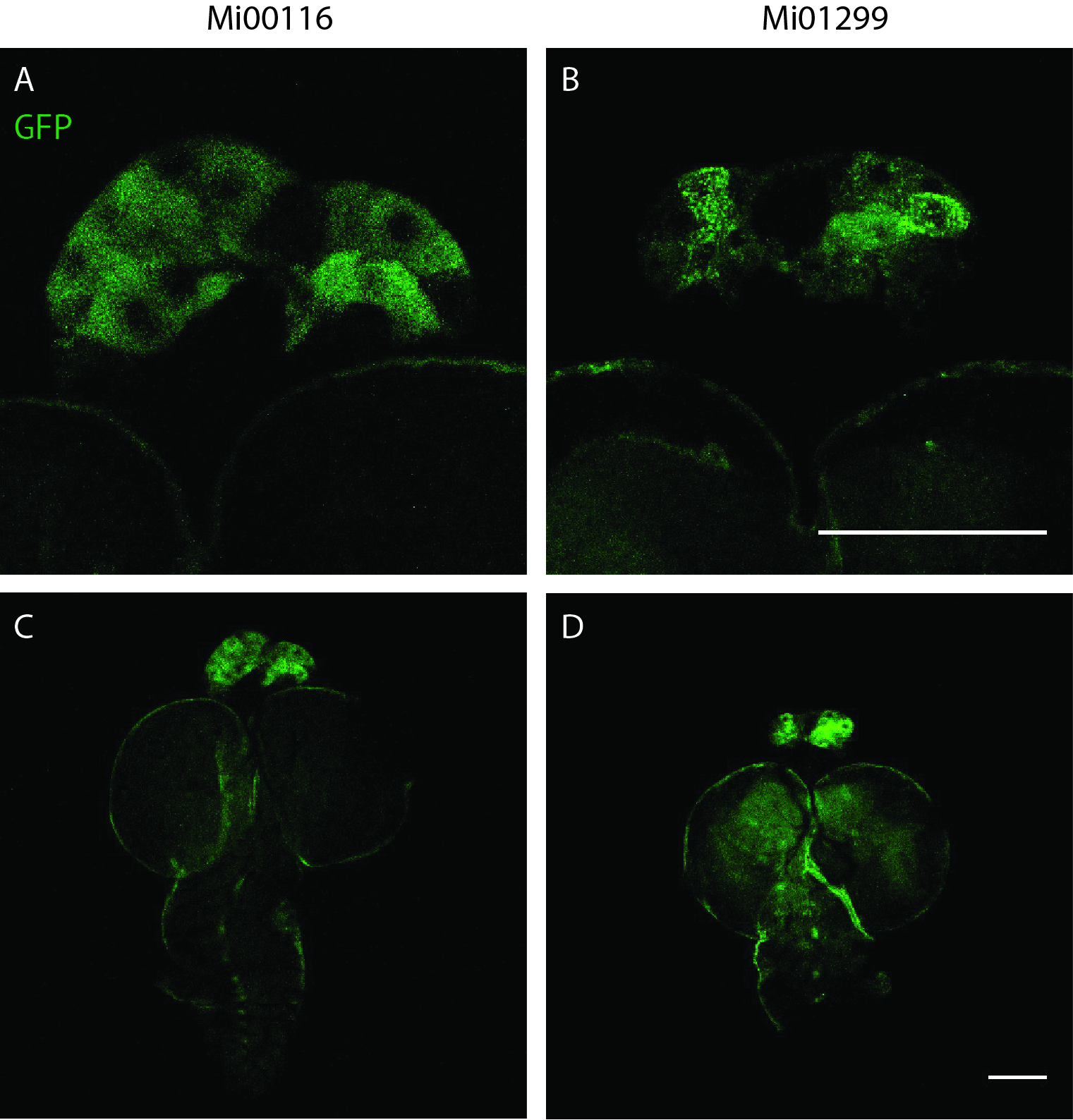
**

**Figure S5** GFP-tagged TepII is enriched in the RG relative to the CNS. RG-CNS complexes were dissected from two GFP-tagged TepII MiMIC lines: Mi00116 and Mi01299 (Nagarkar-Jaiswal *et al.* 2015). Shows live GFP expression in (**A**) Mi00116 RG, (**B**) Mi01299 RG, (**C**) Mi00116 CNS and (**D**) Mi01299 CNS. Scale bars are 100μm.
