# Supplementary material for: Transcriptome Analysis of Drosophila melanogaster Third Instar Larval Ring Glands Points to Novel Functions and Uncovers a Cytochrome p450 Required for Development
Source: G3 (Bethesda). 2016 Dec 13;7(2):467–79. doi: 10.1534/g3.116.037333 (PMC5295594; doi:10.1534/g3.116.037333)
Supplement: Supplementary file 6 [file 467TableS1.docx]

Table S1 *Drosophila melanogaster* lines used in this study

| Name | Origin | Stock # |
| --- | --- | --- |
| Celera (Cel) | BDSC^a^ | 2057 |
| Armenia^14^ (A14) | DGRC^b^ | 103394 |
| Canton-S | BDSC | 64349 |
| *tubulin*-GAL4 | BDSC | 5138 |
| 5’*phm*-GAL4 | Lab | Guittard *et al.* 2011 |
| 5’*6g2*-GAL4 | Lab^c^ | Sztal 2009^d^ |
| *Akh*-GAL4 | BDSC | 25683 |
| UAS-ds*Cyp4g1* | VDRC^e^ | 102864 |
| UAS-ds*Cyp4d2* | VDRC | 103975 |
| UAS-ds*Cyp6g2* | VDRC/Lab | dna3503 |
| UAS-ds*Cyp6u1* | VDRC | 107735 |
| UAS-ds*Cyp6v1* | VDRC | 45584 |
| UAS-EGFP-ban.C | BDSC | 60672 |
| *w*^1118^ | VDRC | 60000 |
| 60100 | VDRC | 60100 |
| UAS-GFP.nls | BDSC | 4775 |
| TepII-GFP-Mi00116 | BDSC | 61650 |
| TepII-GFP-Mi01299 | BDSC | 59402 |

^a^Bloomington Drosophila Stock Centre

^b^Drosophila Genetic Resource Centre

^c^w[1118]; 5’Cyp6g2-GAL4_2b/CyO

^d^Sztal T., 2009 Novel roles of cytochrome p450 genes in insect development. PhD thesis. University of Melbourne, Australia.

^e^Vienna Drosophila Resource Centre, Dietzl *et al.* 2007
