# Supplementary material for: Transcriptome Analysis of Drosophila melanogaster Third Instar Larval Ring Glands Points to Novel Functions and Uncovers a Cytochrome p450 Required for Development
Source: G3 (Bethesda). 2016 Dec 13;7(2):467–79. doi: 10.1534/g3.116.037333 (PMC5295594; doi:10.1534/g3.116.037333)
Supplement: Supplementary file 7 [file 467TableS2.docx]

**Table S2** Primers used in this study

| Primer | Sequence 5’ 🡺 3’ |
| --- | --- |
| CG13220_RT_F | TGGGCAGTGCCTTCTACATTT |
| CG13220_RT_R | CGTACGCACCTCGCTTGTT |
| RpL11_RT_F | CGATCCCTCCATCGGTATCT |
| RpL11_RT_R | AACCACTTCATGGCATCCTC |
| RpL24_qPCR_F | AAATCTACCCCGGTCATGGC |
| RpL24_qPCR_R | CTCGCACTTCTTGTCCAGGA |
| RpL32_RT_F | CCAGTCGGATCGATATGCTAA |
| RpL32_RT_R | GTTCGATCCGTAACCGATGT |
| Cyp4g1_RT_F | TTTGAGGGCCACGATACCAC |
| Cyp4g1_RT_R | CCTTCTGTTCGGCGAAGACT |
| Cyp4d2_RT_F | TGATCTTGGTGAGCTGAAGT |
| Cyp4d2_RT_R | CCCATCGTAAAGTTGGTGCC |
| Cyp6g2_qPCR_F | GAGGTGCTGCGCATGTAT |
| Cyp6g2_qPCR_R | GTTGAGGAAAGTACTGGGGAT |
| Cyp6u1_RT_F | GAGGTGCAAGATCGAACCAG |
| Cyp6u1_RT_R | GAAGGGATGTGGAGTGTGCA |
| Cyp6v1_RT_F | TTCTACTCGCTGAGGCCACA |
| Cyp6v1_RT_R | CCTTGTTCGCCGCACTAAAG |
| Kr-h1_RT_F2 | GAGCTCAATGATGCCGGTC |
| Kr-h1_RT_R2 | GTGGCAGAACTCGCACTC |
