# Supplementary material for: Transcriptome Analysis of Drosophila melanogaster Third Instar Larval Ring Glands Points to Novel Functions and Uncovers a Cytochrome p450 Required for Development
Source: G3 (Bethesda). 2016 Dec 13;7(2):467–79. doi: 10.1534/g3.116.037333 (PMC5295594; doi:10.1534/g3.116.037333)
Supplement: Supplementary file 8 [file 467TableS3.docx]

**Table S3** MIQE table

| **Sample/Template** | **Details** | **Checklist** |
| --- | --- | --- |
| Source | If cancer, was biopsy screened for adjacent normal tissue? | *Drosophila melanogaster* whole 2^nd^ instar larva |
| Method of preservation | Liquid N_2_/RNAlater/formalin | Liquid N_2_ |
| Storage time (if appropriate) | If using samples >6 months old | <2 months old |
| Handling | fresh/frozen/formalin | Frozen |
| Extraction method | TriZol/columns | Reliaprep RNA Cell Miniprep System, Promega OR TriZol (*Cyp6g2* only) |
| RNA: DNA-free | Intron-spanning primers/no RT control | Intron-spanning primers, DNaseI, PCR check |
| Concentration | Nanodrop/ribogreen/microfluidics | Fluorometric quantitation (Qubit) |
| RNA: integrity | Microfluidics/3':5' assay | Agarose gel |
| Inhibition-free | Method of testing | Dilution curve, PCR efficiency <100% |
| **Assay optimisation/validation** |  |  |
| Amplicon details | exon location, amplicon size | *Cyp4g1*: 100bp  *Cyp4d2*: 147bp  *Cyp6u1*: 144bp  *Cyp6v1*: 145bp  *RpL32*: 121bp  *CG13220*: 71bp  *Cyp6g2:* 100bp  *Kr-h1*: 193bp  *RpL11:* 141bp  *RpL24:* 77bp |
| Primer sequence | even if previously published | See **Table S2** |
| *In silico* | BLAST/Primer-BLAST/m-fold | Primer-BLAST, electrophoresis, melt curve |
| empirical | primer concentration/annealing temperature | 10uM, 60deg |
| Priming conditions | oligo-dT/random/combination/target-specific | oligo-dT |
| PCR efficiency | dilution curve | *Cyp4g1*: 1.995  *Cyp4d2*: 2.054  *Cyp6u1*: 1.955  *Cyp6v1*: 1.885  *RpL32*: 1.939  *CG13220*: 1.939  *Cyp6g2:* 2.12  *Kr-h1:* 2.15  *RpL11:* 2.04  *RpL24:* 2.08 |
| Linear dynamic range | spanning unknown targets | 5-fold serial dilution |
| Limits of detection | LOD detection/accurate quantification | *Cyp4g1*: 30.99  *Cyp4d2*: 29.74  *Cyp6u1*: 32.59  *Cyp6v1*: 28.92  *RpL32*: 24.15  *CG13220*: 30.40  *Cyp6g2:* 33.7  *Kr-h1:* 31.03  *RpL11:* 23.36  *RpL24:* 22.47 |
| Intra-assay variation | copy numbers not Cq | *RpL32*: 0.328  *CG13220*: 0.376  *RpL11:* 1.22  *RpL24:* 0.55 |
| **RT/PCR** |  |  |
| Protocols | detailed description, concentrations, volumes | 12.5ul total reaction. 5ul SYBR green Master Mix, 0.25ul F, 0.25ul R, 4.5ul H2O and 2.5ul Template  Initial denaturation: 95deg 5min Denaturation: 95deg 10sec  Annealing: 60deg 30sec  (x40 cycles) |
| Reagents | supplier, Lot number | QuantiFast SYBR green PCR kit, Qiagen |
| Duplicate RT | DCq | Triplicate |
| NTC | Cq & melt curves | CT value or melt curve not detected |
| NAC | DCq beginning:end of qPCR | N/A |
| Positive control | inter-run calibrators | Inter-run calibrator |
| **Data analysis** |  |  |
| Specialist software | e.g., QBAsePlus | qbase+, Biogazelle |
| Statistical justification | e.g., biological replicates | 3 biological replicates for each cross |
| Transparent, validated normalisation | e.g., GeNorm summary | Target and housekeeper validated at the same life stage |
