# Supplementary material for: Transcriptome Analysis of Drosophila melanogaster Third Instar Larval Ring Glands Points to Novel Functions and Uncovers a Cytochrome p450 Required for Development
Source: G3 (Bethesda). 2016 Dec 13;7(2):467–79. doi: 10.1534/g3.116.037333 (PMC5295594; doi:10.1534/g3.116.037333)
Supplement: Supplementary file 9 [file 467TableS4.docx]

**Table S4** Summary of dissections, RNA isolation and RNA-sequencing

| **Sample name** | **Total RNA**  **(ng)** | **RNA QC score (RIN)** | **Gross reads** | **% Reads mapped**  **(overall)** | **% Reads mapped (concordant pairs)** |
| --- | --- | --- | --- | --- | --- |
|  |  |  |  |  |  |
| RG-Cel-A | 850 | 7.3 | 32,415,110 | 90.6 | 86.4 |
| RG-Cel-B | 820 | 7.7 | 28,578,817 | 89.9 | 85.6 |
| RG-Cel-C | 960 | 7.9 | 33,621,275 | 89.8 | 85.1 |
| RG-A14-A | 920 | 7.1 | 29,194,713 | 87.8 | 81.9 |
| RG-A14-B | 1500 | 7.6 | 31,995,041 | 88.4 | 82.5 |
| RG-A14-C | 1800 | 7.5 | 32,937,366 | 87.7 | 81.6 |
| CNS-A^a^ |  |  | 7,249,312 | 91.4 | 86.7 |
| CNS-B^a^ |  |  | 23,628,510 | 87.4 | 78.6 |

^a^Reads downloaded from modMINE accession: SRX029398 (Contrino 2012)
