# Supplementary material for: Transcriptome Analysis of Drosophila melanogaster Third Instar Larval Ring Glands Points to Novel Functions and Uncovers a Cytochrome p450 Required for Development
Source: G3 (Bethesda). 2016 Dec 13;7(2):467–79. doi: 10.1534/g3.116.037333 (PMC5295594; doi:10.1534/g3.116.037333)
Supplement: Supplementary file 10 [file 467TableS5.docx]

**Table S5** Most abundant genes in the ring gland, sorted by Celera FPKM

| **Flybase symbol** | **Gene name** | **Celera**  **FPKM** | **Fold Enrichment^a^** | **Armenia^14^**  **FPKM** | **Biological Process^b^** |
| --- | --- | --- | --- | --- | --- |
|  |  |  |  |  |  |
| *RpL41* | *Ribosomal protein L41* | 21,415 | +1.53 | 20,998 | *translation* |
| *phm* | *Phantom* | 13,305 | +113.35 | 15,436 | ecdysone biosynthetic process |
| *sad* | *Shadow* | 12,617 | +161.98 | 16,483 | ecdysone biosynthetic process |
| *RpL39* | *Ribosomal protein L39* | 9,855 | +6.72 | 10,531 | *translation* |
| *Ef1α48D* | *Elongation factor 1α48D* | 6,629 | +2.21 | 5,763 | *translation* |
| *CG10970* |  | 5,966 | +62.90 | 6,178 |  |
| *Npc1a* | *Niemann-Pick type C-1a* | 5,479 | +113.67 | 5,228 | regulation of cholesterol transport |
| *Qm* | *Quemao* | 4,433 | +8.21 | 4,190 | *isoprenoid biosynthetic process* |
| *RpS27A* | *Ribosomal protein S27A* | 4,249 | +1.68 | 3,995 | *translation* |
| *Thor* | *Thor* | 4,239 | +46.91 | 3,887 | anitbacterial humoral response |
| *Act5C* | *Actin 5C* | 4,117 | -1.84 | 4,668 | mitotic cytokinesis |
| *RpS16* | *Ribosomal protein S16* | 4,050 | +3.58 | 3,614 | *translation* |
| *RpL38* | *Ribosomal protein L38* | 4,001 | +6.21 | 3,961 | *translation* |
| *RpLP1* | *Ribosomal protein LP1* | 3,924 | +1.55 | 3,465 | *translation* |
| *Hsp27* | *Heat shock protein 27* | 3,844 | -1.25 | 3,017 | response to stress |
| *RpL29* | *Ribosomal protein L29* | 3,823 | +3.83 | 3,692 | *translation* |
| *RpS3A* | *Ribosomal protein S3A* | 3,817 | +5.81 | 3,754 | *translation* |
| *RpL23* | *Ribosomal protein L23* | 3,770 | +2.08 | 3,415 | *translation* |
| *nvd* | *Neverland* | 3,759 | +194.61 | 3,448 | ecdysteroid biosynthetic process |
| *CG15919* |  | 3,706 | +3,370.16 | 5,944 |  |
| *RpS28B* | *Ribosomal protein S28B* | 3,624 | +3.93 | 3,503 | *translation* |
| *RpS9* | *Ribosomal protein S9* | 3,521 | +1.76 | 2,786 | *translation* |
| *RpS7* | *Ribosomal protein S7* | 3,518 | +1.16 | 3,283 | *translation* |
| *RpL36A* | *Ribosomal protein L36A* | 3,500 | +4.66 | 3,137 | *translation* |
| *RpL5* | *Ribosomal protein L5* | 3,458 | +3.92 | 3,173 | *translation* |
| *RpS15* | *Ribosomal protein S15* | 3,398 | -1.40 | 3,208 | *translation* |
| *RpS25* | *Ribosomal protein S25* | 3,387 | +1.53 | 2,977 | *translation* |
| *RpS30* | *Ribosomal protein S30* | 3,369 | +2.70 | 3,154 | *translation* |
| *RpLP2* | *Ribosomal protein LP2* | 3,363 | +1.67 | 3,308 | *translation* |
| *RpS8* | *Ribosomal protein S8* | 3,262 | +1.68 | 2,887 | neurogenesis |
| *RpS29* | *Ribosomal protein S29* | 3,240 | +1.29 | 2,938 | neurogenesis |
| *CG4408* |  | 3,221 | +123.63 | 1,986 | *proteolysis* |
| *RpL21* | *Ribosomal protein L21* | 3,213 | -1.77 | 2,925 | *translation* |
| *RpS4* | *Ribosomal protein S4* | 3,195 | +4.12 | 3,160 | *translation* |
| *RpS13* | *Ribosomal protein S13* | 3,182 | +2.06 | 2,910 | *translation* |
| *RpL27A* | *Ribosomal protein L27A* | 3,169 | +2.24 | 2,966 | *translation* |
| *RpS20* | *Ribosomal protein S20* | 3,140 | -1.08 | 2,758 | *translation* |
| *RpS5a* | *Ribosomal protein S5a* | 3,092 | +2.21 | 2,609 | *translation* |

^a^Fold enrichment in the Celera ring gland versus the central nervous system.

^b^Regular text = based on experimental evidence, italics = based on predictions or assertions

We have selected GO terms that were most informative for our study, other GO terms for each gene can be found at FlyBase (St Pierre *et al.* 2014)
