# Supplementary material for: Transcriptome Analysis of Drosophila melanogaster Third Instar Larval Ring Glands Points to Novel Functions and Uncovers a Cytochrome p450 Required for Development
Source: G3 (Bethesda). 2016 Dec 13;7(2):467–79. doi: 10.1534/g3.116.037333 (PMC5295594; doi:10.1534/g3.116.037333)
Supplement: Supplementary file 11 [file 467TableS6.docx]

**Table S6** Genes with the most significant differential expression between the Celera ring gland and the Armenia^14^ ring gland

| **Flybase symbol** | **Gene name** | **FPKM** | **Fold**  **Enrichment** | **GO term^a^** | |  |
| --- | --- | --- | --- | --- | --- | --- |
|  |  |  |  | **Biological Process** | | **Molecular Function** |
| **Genes enriched in the Celera ring gland** | | | | |  | |
| *CG42249* |  | 256 | +623.28 | *nucleotide catabolic process* | nucleoside-diphosphatase activity | |
| *CG9394* |  | 230 | +1,122.03 | *lipid metabolic process* | *phosphoric diester hydrolase activity* | |
| *Obp56a* | *Oderant-binding protein 56a* | 140 | +31.79 | sensory perception of smell | *oderant binding* | |
| *CG31477* |  | 89 | +70.73 | *ATP synthesis coupled proton transport* | *hydrogen-exporting ATPase activity* | |
| *CG30148* |  | 60 | +105.72 |  |  | |
| *CG8539* |  | 59 | +61.98 | *proteolysis* | *metallocarboxypeptidase activity* | |
| *CG31231* |  | 20 | +360.47 |  |  | |
| *Ir76a* | *Ionotropic receptor 76a* | 12 | +64.27 | detection of chemical synthesis involved in sensory perception | olfactory receptor activity | |
| *Ir93a* | *Ionotropic receptor 93a* | 10 | +61.93 | detection of chemical synthesis involved in sensory perception | *ionotropic glutamate receptor activity* | |
| *CG18853* |  | 9 | +8,673.25 | *DNA repair* | *deoxyribodipyrimidine photo-lyase activity* | |
| *CG6023* |  | 3 | +5,455.80 |  |  | |
| **Genes enriched in the Armenia^14^ ring gland** | | | | |  | |
| *CG13728* |  | 294 | +141.59 |  |  | |
| *Prat2* | *Phosphoribosylamidotransferase 2* | 173 | +47.02 | *‘de novo’ IMP biosynthetic process* | *amidophosphoribosyl-transferase activity* | |
| *CG13731* |  | 148 | +52.93 |  |  | |
| *CG7017* |  | 59 | +30.44 | *chitin metabolic process* | *chitin binding* | |
| *CG11697* |  | 54 | +255.03 |  |  | |
| *CG9021* |  | 53 | +58.21 |  |  | |
| *LysB* | *Lysozyme B* | 41 | +100.36 | *antimicrobial humoral response* | *lysozyme activity* | |
| *Muc26B* | *Mucin 26b* | 36 | +54.48 | chitin metabolism |  | |
| *LysC* | *Lysozyme C* | 25 | +48.51 | lysozyme activity |  | |
| *CG9815* |  | 20 | +37.39 |  |  | |
| *CG11294* |  | 6 | +44.05 | *regulation of transcription* | *transcription factor activity* | |
| *Tsp42Eb* | *Tetraspanin 42eb* | 6 | +31.66 |  | *tetraspanin domain* | |
| *ric8b* |  | 4 | +4,129.46 |  |  | |
| *CG17105* |  | 4 | +4,098.42 |  |  | |

^a^Regular text = based on experimental evidence, italics = based on predictions or assertions

We have selected GO terms that were most informative for our study, other GO terms for each gene can be found at FlyBase (St Pierre *et al.* 2014)
