# Supplementary material for: Transcriptome Analysis of Drosophila melanogaster Third Instar Larval Ring Glands Points to Novel Functions and Uncovers a Cytochrome p450 Required for Development
Source: G3 (Bethesda). 2016 Dec 13;7(2):467–79. doi: 10.1534/g3.116.037333 (PMC5295594; doi:10.1534/g3.116.037333)
Supplement: Supplementary file 12 [file 467TableS7.docx]

**Table S7** Most highly enriched genes in the ring gland (A14 data)

| **Flybase symbol** | **Gene name** | **FPKM^a^** | **Fold Enrichment^a^** | **GO Term^b^** | |
| --- | --- | --- | --- | --- | --- |
|  |  |  |  | **Biological process** | **Molecular function** |
| *phm* | *Phantom* | 15,436 | +131.50 | ecdysone biosynthetic process | ecdysteroid 25-hydroxylase activity |
| *sad* | *Shadow* | 16,483 | +211.62 | ecdysone biosynthetic process | ecdysteroid 2-hydroxylase activity |
| *Npc1a* | *Niemann-Pick C type 1a* | 5,228 | +108.46 | regulation of cholesterol transport | *hedgehog receptor activity* |
| *nvd* | *Neverland* | 3,448 | +178.50 | ecdysteroid biosynthetic process | *oxidoreductase activity* |
| *CG15919* |  | 5,944 | +54,054 |  |  |
| *CG4408* |  | 1,986 | +76.22 | *proteolysis* | *metallocarboxypeptidase activity* |
| *CG6310* |  | 1,774 | +156.90 |  |  |
| *nobo* | *Noppera-bo* | 2,938 | +310.19 | ecdysteroid biosynthetic process | glutathione transferase activity |
| *Cyp6g2* | *Cytochrome p450 6g2* | 740 | +108.25 | oxidation-reduction process | *monoxygenase activity* |
| *dib* | *Disembodied* | 1,717 | +386.45 | *ecdysone biosynthetic process* | ecdysteroid 22-hydroxylase activity |
| *CG10337* |  | 878 | +211.61 |  |  |
| *CG9184* |  | 27 | +6.89 |  |  |
| *jhamt* | *Juvenile hormone acid methyltransferase* | 691 | +480.49 | juvenile hormone biosynthetic process | juvenile hormone acid methyltransferase activity |
| *CG4822* |  | 506 | +116.38 |  | *transporter activity* |
| *CG6426* |  | 193 | +47.98 | multicellular organism reproduction | *lysozyme activity* |
| *CG13101* |  | 430 | +202.16 |  |  |
| *Tsp42El* | *Tetraspanin 42El* | 484 | +155.44 |  |  |
| *CG2254* |  | 330 | +90.46 | *metabolic process* | *oxidoreductase activity* |
| *Lectin-galC1* | *Galactose-specific C-type lectin* | 216 | +182.72 | induction of bacterial agglutination | galactose binding |
| *tor* | *Torso* | 134 | +84.01 | metamorphosis | protein tyrosine kinase activity |
| *CG30471* |  | 241 | +533.01 |  | *transferase activity* |
| *CG40006* |  | 5 | +9.94 | cell adhesion |  |
| *Cyp6a13* | *Cytochrome p450 6a13* | 4 | +20.42 | defense response to bacterium | *oxidoreductase activity* |

^a^A14 data are provided here, for Cel data see **Table 1**

^b^Regular text = based on experimental evidence, italics = based on predictions or assertions

We have selected GO terms that were most informative for our study, other GO terms for each gene can be found at FlyBase (St Pierre *et al.* 2014)
