# Supplementary material for: Transcriptome Analysis of Drosophila melanogaster Third Instar Larval Ring Glands Points to Novel Functions and Uncovers a Cytochrome p450 Required for Development
Source: G3 (Bethesda). 2016 Dec 13;7(2):467–79. doi: 10.1534/g3.116.037333 (PMC5295594; doi:10.1534/g3.116.037333)
Supplement: Supplementary file 13 [file 467TableS8.docx]

**Table S8** Expression of select genes involved in ecdysteroidogenesis (A14 data)

| **Flybase Symbol** | **Gene name** | **FPKM^a^** | **Fold**  **Enrichment^a^** | **q-value** |
| --- | --- | --- | --- | --- |
| **Ecdysteroidogenic enzymes** | | | |  |
| *nobo* | *Noppera-bo* | 2,938 | +310.19 | <0.001 |
| *nvd* | *Neverland* | 3,448 | +178.50 | <0.001 |
| *spo* | *Spook* | 1 | +16.45 | 0.6 |
| *spok* | *Spookier* | 0.0^b^ | 0 | 1 |
| *sro* | *Shroud* | 1,021 | +78.82 | <0.001 |
| *phm* | *Phantom* | 15,436 | +131.50 | <0.001 |
| *dib* | *Disembodied* | 1,717 | +386.45 | <0.001 |
| *sad* | *Shadow* | 16,483 | +211.62 | <0.001 |
| *shd* | *Shade* | 1 | +2.21 | 0.2 |
| **Cholesterol homeostasis** | | | |  |
| *Npc1a* | *Niemann Pick C type 1a* | 5,228 | +108.46 | <0.001 |
| *Npc2a* | *Niemann Pick C type 2a* | 105 | -1.30 | 0.07 |
| *Start1* | *Start1* | 2,960 | +117.62 | <0.001 |
| *mdy* | *Midway* | 125 | +25.31 | <0.001 |
| **PTTH signalling** | | | |  |
| *tor* | *Torso* | 134 | +84.01 | <0.001 |
| *Ras* | *Ras* | 89 | +1.78 | 0.01 |
| *Raf* | *Raf* | 12 | -1.92 | <0.001 |
| *ERK* | *ERK* | 0.0^b^ | 0 | 1 |
| *Cam* | *Calmodulin* | 918 | +1.61 | <0.001 |
| *rut* | *rutabega* | 6 | -8.84 | <0.001 |
| *PKA* | *Protein kinase A* | 48 | -2.80 | <0.001 |
| *RpS6* | *Ribosomal protein S6* | 2,226 | +1.17 | 0.3 |
| *Hr4* | *Hormone receptor 4* | 7 | -2.13 | <0.001 |
| **Insulin signalling** | | | |  |
| *InR* | *Insulin receptor* | 8 | -2.23 | <0.001 |
| *Pi3K* | *Phosphotidylinositol 3 kinase* | 21 | +1.15 | 0.4 |
| *Akt* | *Akt* | 51 | +1.44 | 0.002 |
| **Activin signalling** | | | |  |
| *babo* | *Baboon* | 47 | -1.13 | 0.4 |
| *put* | *punt* | 71 | +3.23 | <0.001 |
| *smad2/smox* | *Smad on X* | 43 | -2.53 | <0.001 |
| **Nitric oxide signalling** | | | |  |
| *E75* | *Ecdysone-induced protein 75* | 66 | -3.41 | <0.001 |
| *Hr46* | *Hormone receptor-like 46* | 17 | +2.03 | <0.001 |
| *ftz-f1* | *ftz transcription factor 1* | 3 | -1.53 | 0.04 |
| **TOR signalling** | | | |  |
| *TSC1* | *TSC1* | 21 | -1.32 | 0.03 |
| *TSC2/gig* | *TSC2* | 15 | +1.32 | 0.3 |
| *Tor* | *Target of rapamycin* | 17 | -1.28 | 0.05 |
| **Serotonin signalling** | | | |  |
| *5-HT1A* | *5-hydroxytryptamine (serotonin) receptor 1A* | 2 | -4.71 | <0.001 |
| **JH signalling** | | | |  |
| *Met* | *Methoprene-tolerant* | 4 | -2.76 | <0.001 |
| *gce* | *Germ cell-expressed bHLH-PAS* | 5 | -2.06 | 0.001 |
| **20E signalling** |  |  |  |  |
| *EcR* | *Ecdysone receptor* | 110 | +2.19 | <0.001 |
| *usp* | *Ultraspiracle* | 29 | -1.84 | 0.02 |

^a^A14 data are provided here, for Cel data see **Table 2**

^b^Genes located in heterochromatic regions were not included in reference genome. Reads corresponding to these genes were therefore not aligned by Tophat, hence the 0.0 FPKM score.
