# Supplementary material for: Transcriptome Analysis of Drosophila melanogaster Third Instar Larval Ring Glands Points to Novel Functions and Uncovers a Cytochrome p450 Required for Development
Source: G3 (Bethesda). 2016 Dec 13;7(2):467–79. doi: 10.1534/g3.116.037333 (PMC5295594; doi:10.1534/g3.116.037333)
Supplement: Supplementary file 14 [file 467TableS9.docx]

**Table S9** Ring gland-enriched cytochrome p450 genes (A14 data)

| **cytochrome p450** | **FPKM^a^** | **Fold**  **Enrichment^a^** | **q value** | **Annotated Biological Process^b^** | **Ubiquitous RNAi knockdown^c^** | **Clade stability across *Drosophila* species^d^** |
| --- | --- | --- | --- | --- | --- | --- |
|  |  |  |  |  |  |  |
| *phm* | 15,436 | +131.50 | <0.001 | ecdysone biosynthetic process | Lethal | Stable |
| *sad* | 16,483 | +211.62 | <0.001 | ecdysone biosynthesis process | n/a | Stable |
| *Cyp6g2* | 740 | +108.25 | <0.001 | *oxidation-reduction process* | Lethal | Stable |
| *dib* | 1,717 | +386.45 | <0.001 | ecdysone biosynthetic process | n/a | Stable |
| *Cyp6a13* | 4 | +20.42 | <0.001 | defence response to bacterium | Viable | Gene loss |
| *Cyp6v1* | 56 | +4.93 | <0.001 | *oxidation-reduction process* | n/a | Stable |
| *Cyp12e1* | 75 | +12.89 | <0.001 | *oxidation-reduction process* | Viable | Gene gain |
| *Cyp310a1* | 22 | +33.52 | <0.001 | negative regulation of Wnt signaling pathway | n/a | Gene loss |
| *Cyp6u1* | 33 | +3.24 | <0.001 | *oxidation-reduction process* | n/a | Stable |
| *Cyp9f2* | 22 | +2.04 | <0.001 | wing disc development | Viable | Gene gain |
| *Cyp4g1* | 20 | +2.92 | 0.3 | lipid metabolic process | Lethal | Stable |
| *Cyp303a1* | 15 | +17.22 | <0.001 | sensory organ development | n/a | Stable |
| *Cyp4d2* | 73 | +17.73 | <0.001 | *oxidation-reduction process* | Lethal | Gene loss |
| *Cyp6d4* | 16 | +2.88 | <0.001 | wing disc development | Viable | Gene gain |
| *Cyp18a1* | 4 | +17.10 | <0.001 | ecdysteroid catabolic process | Lethal | Stable |
|  |  |  |  |  |  |  |

^a^Only A14 RG data are provided here, for Cel data see **Table 3**

^b^Regular text = based on experimental evidence, italics = based on predictions or assertions

We have selected GO terms that were most informative for our study, other GO terms for each gene can be found at FlyBase (St Pierre *et al.* 2014)

^c^Chung *et al.* 2009, Guittard *et al.* 2011, Qiu *et al.* 2012

^d^Good *et al.* 2014
