# Supplementary material for: Transcriptome Analysis of Drosophila melanogaster Third Instar Larval Ring Glands Points to Novel Functions and Uncovers a Cytochrome p450 Required for Development
Source: G3 (Bethesda). 2016 Dec 13;7(2):467–79. doi: 10.1534/g3.116.037333 (PMC5295594; doi:10.1534/g3.116.037333)
Supplement: Supplementary file 15 [file 467TableS10.docx]

**Table S10** Subset of 288 genes with significant enrichment >10-fold in both the Celera ring gland and the Armenia^14^ ring gland, relative to the CNS. Significant D-scores^a^ noted.

| **Flybase Symbol** | **D**  **score^a^** | **Flybase Symbol** | **D**  **score^a^** | **Flybase Symbol** | **D**  **score^a^** | **Flybase Symbol** | **D**  **score^a^** | **Flybase Symbol** | **D**  **score^a^** | **Flybase Symbol** | **D**  **score^a^** |
| --- | --- | --- | --- | --- | --- | --- | --- | --- | --- | --- | --- |
| *Adgf-C* | 0.8 | *CG13516* |  | *CG30438* | 0.9 | *CG5104* |  | *Cyp28a5* |  | *Ncc69* |  |
| *Ag5r2* | 0.9 | *CG13603* |  | *CG30471* | 0.9 | *CG5112* | 0.6 | *Cyp303a1* | 0.7 | *nimB4* | 0.9 |
| *AGO3* |  | *CG13717* |  | *CG31145* |  | *CG5278* |  | *Cyp310a1* | 0.6 | *nimC3* | 0.8 |
| *α-Est5* |  | *CG13822* | 0.9 | *CG31146* |  | *CG5335* |  | *Cyp312a1* |  | *nocturnin* |  |
| *αTub85E* |  | *CG13992* | 0.8 | *CG31370* |  | *CG5381* |  | *Cyp6a13* |  | *Npc1a* | 0.7 |
| *Arpc3B* |  | *CG14075* | 0.8 | *CG31436* |  | *CG5431* |  | *Cyp6g2* |  | *Nplp2* | 0.9 |
| *Atet* |  | *CG14107* | 0.8 | *CG31689* |  | *CG5577* |  | *dib* |  | *nvd* |  |
| *blot* |  | *CG14253* |  | *CG31777* | 0.8 | *CG5599* |  | *drd* | 0.9 | *Oatp74D* |  |
| *bond* |  | *CG14301* | 0.7 | *CG31789* | 0.9 | *CG5773* | 0.9 | *Drs* | 0.8 | *olf186-M* |  |
| *bru-2* |  | *CG1443* |  | *CG31937* |  | *CG6232* | 0.8 | *eater* | 0.9 | *Pbgs* |  |
| *CAH2* | 0.9 | *CG14899* |  | *CG31955* |  | *CG6310* |  | *Ent1* |  | *PebIII* | 0.8 |
| *Cda4* | 0.6 | *CG14964* |  | *CG32280* |  | *CG6426* | 0.9 | *fbp* |  | *pgant4* |  |
| *CG10026* |  | *CG1499* | 0.9 | *CG32365* |  | *CG6643* |  | *Fkbp13* | 0.8 | *Pgd* |  |
| *CG10232* | 0.7 | *CG15046* | 0.8 | *CG32594* |  | *CG6680* |  | *fus* |  | *PH4αMP* | 0.8 |
| *CG10280* |  | *CG15083* |  | *CG3267* |  | *CG7091* |  | *Gclc* |  | *phm* |  |
| *CG10311* |  | *CG15098* |  | *CG33225* | 0.8 | *CG7272* |  | *gk* |  | *pirk* |  |
| *CG10337* |  | *CG15347* | 0.8 | *CG33459* |  | *CG7497* |  | *Hand* |  | *Pkg21D* |  |
| *CG10399* |  | *CG15611* |  | *CG33460* | 0.6 | *CG7510* |  | *He* | 0.8 | *ppk16* |  |
| *CG10444* |  | *CG15695* |  | *CG33461* | 0.7 | *CG7759* |  | *Hmgs* |  | *Ppt1* | 0.6 |
| *CG10527* |  | *CG15709* |  | *CG33465* | 0.8 | *CG7966* |  | *ImpE3* | 0.8 | *Proc-R* |  |
| *CG10650* | 0.7 | *CG1575* |  | *CG3355* | 0.9 | *CG8008* |  | *ImpL2* | 0.9 | *psh* | 0.9 |
| *CG10663* | 0.6 | *CG15786* |  | *CG33970* |  | *CG8046* |  | *IP3K1* |  | *pst* |  |
| *CG10764* | 0.8 | *CG15887* | 0.9 | *CG34250* |  | *CG8083* |  | *Ir41a* |  | *pyd3* |  |
| *CG10908* |  | *CG15917* | 0.8 | *CG34446* |  | *CG8090* |  | *jhamt* |  | *Ret* | 0.5 |
| *CG1092* | 0.8 | *CG15919* |  | *CG3502* | 0.9 | *CG8239* |  | *Jheh1* |  | *Rpt6R* |  |
| *CG10932* |  | *CG16799* |  | *CG3868* | 0.9 | *CG8306* |  | *Jon25Biii* | 0.8 | *sad* |  |
| *CG11103* |  | *CG16820* | 0.8 | *CG40006* |  | *CG8317* | 0.9 | *Jon66Cii* | 0.8 | *spz5* | 0.5 |
| *CG11137* |  | *CG17018* |  | *CG40127* |  | *CG8331* |  | *Jon99Ciii* |  | *Start1* |  |
| *CG1124* | 0.7 | *CG17323* | 0.7 | *CG40439* |  | *CG8596* |  | *Jon99Fi* | 0.9 | *sut1* |  |
| *CG11370* | 0.8 | *CG17633* | 0.9 | *CG4096* | 0.8 | *CG8630* |  | *Jon99Fii* | 0.8 | *t* |  |
| *CG11438* |  | *CG17646* |  | *CG4151* | 0.8 | *CG8646* | 0.9 | *jp* |  | *TepI* | 0.8 |
| *CG11665* |  | *CG17721* |  | *CG4213* |  | *CG8664* | 0.8 | *kni* |  | *TepII* | 0.8 |
| *CG11762* |  | *CG17919* | 0.8 | *CG42345* |  | *CG8701* |  | *knrl* |  | *Thor* |  |
| *CG11836* |  | *CG17928* |  | *CG42369* | 0.8 | *CG8738* | 0.8 | *l(2)03659* |  | *tim* |  |
| *CG12009* | 0.9 | *CG18278* | 0.8 | *CG4259* | 0.8 | *CG8788* |  | *l(2)08717* |  | *tin* |  |
| *CG12026* |  | *CG18594* |  | *CG4267* |  | *CG9095* | 0.9 | *lace* |  | *tor* | 0.5 |
| *CG12063* | 0.8 | *CG1941* |  | *CG4386* | 0.9 | *CG9171* |  | *LamC* |  | *trp* |  |
| *CG12068* |  | *CG2118* |  | *CG4408* | 0.5 | *CG9184* | 0.7 | *lbm* |  | *tsl* | 0.8 |
| *CG12811* | 0.9 | *CG2135* | 0.8 | *CG4572* | 0.7 | *CG9372* | 0.8 | *lin-28* |  | *Tsp42Ef* |  |
| *CG12825* |  | *CG2254* |  | *CG4594* |  | *CG9505* | 0.8 | *LysS* | 0.9 | *Tsp42Eg* |  |
| *CG13003* | 0.9 | *CG2837* | 0.8 | *CG4666* |  | *CG9541* |  | *mdy* |  | *Tsp42Ei* |  |
| *CG13028* | 0.5 | *CG30000* |  | *CG4680* |  | *CG9917* |  | *meso18E* |  | *Tsp42Ek* |  |
| *CG13035* |  | *CG30046* | 0.8 | *CG4688* |  | *CG9989* | 0.8 | *MSBP* |  | *Tsp42El* |  |
| *CG13045* |  | *CG30104* | 0.8 | *CG4716* | 0.9 | *CHKov1* |  | *msta* |  | *Ugt36Bc* | 0.8 |
| *CG1309* |  | *CG30154* | 0.9 | *CG4793* | 0.8 | *CHKov2* |  | *mth* | 0.8 | *Ugt86Di* | 0.6 |
| *CG13101* |  | *CG30194* |  | *CG4822* |  | *Cng* |  | *mthl2* | 0.8 | *wdp* | 0.9 |
| *CG1319* |  | *CG30285* | 0.9 | *CG4927* | 0.9 | *Cyp12e1* |  | *mthl3* | 0.9 | *yellow-b* | 0.9 |
| *CG13335* |  | *CG30427* |  | *CG4950* | 0.8 | *Cyp18a1* |  | *mthl6* | 0.9 | *yellow-f* | 0.7 |

^a^SignalP 4.1 (Petersen *et al.* 2011), genes with a D-score >0.45 are predicted to possess a signal peptide.
