# Supplementary material for: Transcriptome Analysis of Drosophila melanogaster Third Instar Larval Ring Glands Points to Novel Functions and Uncovers a Cytochrome p450 Required for Development
Source: G3 (Bethesda). 2016 Dec 13;7(2):467–79. doi: 10.1534/g3.116.037333 (PMC5295594; doi:10.1534/g3.116.037333)
Supplement: Supplementary file 16 [file 467TableS11.docx]

**Table S11** Top ring gland-enriched transcripts from Ou *et al* (2016), sorted according to enrichment level in present study.

| **Present** and **significantly enriched** in ring gland | | | | | | | **107 genes** | |
| --- | --- | --- | --- | --- | --- | --- | --- | --- |
| *Acph-1*  *AGO3*  *Akh*  *alt*  *Atet*  *CG10171*  *CG10280*  *CG11762*  *CG12290*  *CG12880*  *CG13045*  *CG13101*  *CG13506*  *CG14075* | *CG14107*  *CG14372*  *CG15046*  *CG15201*  *CG15550*  *CG15919*  *CG30471*  *CG30427*  *CG31145*  *CG32573*  *CG33156*  *CG33465*  *CG33970*  *CG34216* | *CG34263*  *CG42534*  *CG4678*  *CG4681*  *CG4716*  *CG4822*  *CG5278*  *CG6163*  *CG6201*  *CG6650*  *CG6962*  *CG7458*  *CG7497*  *CG7510* | *CG7730*  *CG8239*  *CG8630*  *CG9184*  *CG9541*  *CG9593*  *Cng*  *curled*  *Cyp303a1*  *Cyp6a13*  *dib*  *Eb1*  *Eip63E*  *Ext2* | *fdx2*  *form3*  *frayed*  *Gagr*  *Gclc*  *GluRIIB*  *Hand*  *Heix*  *Hs6st*  *ImpE3*  *Ir41a*  *jhamt*  *Kaz1*  *Kif3C* | *Lst8*  *loh*  *mab-21*  *Mes2*  *mld*  *mthl12*  *mthl6*  *mthl7*  *nvd*  *Oatp74D*  *pdm3*  *phm*  *Plc21C*  *ppk18* | *Proc-R*  *punch*  *Pvf2*  *sad*  *sevenless*  *shakB*  *side*  *Snap25*  *spz5*  *sro*  *Sr-CIII*  *star1*  *timeless*  *tinman* | | *torso*  *Traf4*  *TrpA1*  *VhaSFD*  *vvl*  *wdp*  *wts* |
| **Present** and **significantly depleted** in ring gland | | | | | | | **3 genes** | |
| *CG9813*  *ImpE1*  *smi35A* |  |  |  |  |  |  | |  |
| **Absent** (FPKM<1) | | | | | | | **26 genes** | |
| *CG11113*  *CG11229*  *CG11437*  *CG12278* | *CG14069*  *CG1756*  *CG18294*  *CG30395* | *CG33557*  *CG34051*  *CG34217* | *CG40485*  *Cyp28c1*  *dpr6* | *Hsp70Ab*  *Hsp70Ba*  *Hsp70Bbb* | *Hsp70Bc*  *MstProx*  *mthl13* | *SIFR*  *snail*  *SNCF* | | *Toll-4*  *TwdIS*  *Ugt37c1* |
