# Supplementary material for: Transcriptome Analysis of Drosophila melanogaster Third Instar Larval Ring Glands Points to Novel Functions and Uncovers a Cytochrome p450 Required for Development
Source: G3 (Bethesda). 2016 Dec 13;7(2):467–79. doi: 10.1534/g3.116.037333 (PMC5295594; doi:10.1534/g3.116.037333)
Supplement: Supplementary file 17 [file 467TableS12.docx]

**Table S12** Ring gland-enriched serine proteases (A14 data)

| **Flybase symbol** | **Gene name** | **FPKM^a^** |
| --- | --- | --- |
| *CG4572* |  | 564 |
| *Jon99Cii* | *Jonah 99Cii* | 5 |
| *CG33465* |  | 36 |
| *CG33460* |  | 24 |
| *CG9372* |  | 40 |
| *CG4259* |  | 41 |
| *CG15046* |  | 28 |
| *CG10663* |  | 18 |
| *CG10232* |  | 17 |
| *CG4793* |  | 13 |
| *CG4927* |  | 7 |
| *CG4386* |  | 28 |
| *Jon99Fii* | *Jonah 99Fii* | 4 |
| *CG10764* |  | 13 |
| *Jon25Biii* | *Jonah 25Biii* | 6 |
| *CG3355* |  | 11 |
| *Jon99Fi* | *Jonah 99Fi* | 3 |
| *CG33225* |  | 2 |
| *CG33461* |  | 3 |
| *Jon66Cii* | *Jonah 66Cii* | 2 |
| *CG8738* |  | 3 |
| *psh* | *Persephone* | 6 |

^a^Only A4 RG data are provided here, for Cel data see **Table 5**
