# Supplementary material for: Transcriptome Analysis of Drosophila melanogaster Third Instar Larval Ring Glands Points to Novel Functions and Uncovers a Cytochrome p450 Required for Development
Source: G3 (Bethesda). 2016 Dec 13;7(2):467–79. doi: 10.1534/g3.116.037333 (PMC5295594; doi:10.1534/g3.116.037333)
Supplement: Supplementary file 18 [file 467TableS13.docx]

**Table S13** Ring gland expression of key genes in the immune response pathways (A14 data)

| **Flybase symbol** | **Gene name** | **FPKM^a^** | **Fold Enrichment^a^** | **q-value** |
| --- | --- | --- | --- | --- |
| **Toll Pathway** |  |  |  |  |
| *Tl* | *Toll* | 62 | +1.64 | <0.001 |
| *Myd88* | *Myd88* | 19 | +2.21 | 0.09 |
| *pll* | *Pelle* | 18 | +1.87 | 0.8 |
| *tub* | *Tube* | 33 | +1.04 | <0.001 |
| *cact* | *Cactus* | 239 | +4.45 | <0.001 |
| *Dif* | *Dorsal-related immunity factor* | 33 | +3.33 | <0.001 |
| *Drs* | *Drosomycin* | 30 | +10.48 | <0.001 |
| **Imd Pathway** |  |  |  |  |
| *PGRP-LC* | *Peptidoglycan recognition protein LC* | 8 | +4.84 | <0.001 |
| *imd* | *Immune deficiency* | 22 | +1.73 | 0.6 |
| *Fadd* | *Fas-associated death domain orthologue* | 14 | +2.76 | 0.1 |
| *Dredd* | *Death related ced-3* | 46 | +4.29 | <0.001 |
| *Tak1* | *TGF-β activated kinase 1* | 26 | -1.44 | 0.004 |
| *key* | *Kenny* | 46 | +1.13 | 0.5 |
| *ird5* | *Immune response deficient 5* | 17 | +5.13 | <0.001 |
| *Rel* | *Relish* | 34 | +2.32 | <0.001 |
| *DptB* | *Diptericin* | 1 | +1.78 | 1 |

^a^Unless otherwise stated, only A14 RG data are provided here. For Cel data see **Table 6**
